# Mitochondrial DNA from El Mirador Cave (Atapuerca, Spain) Reveals the Heterogeneity of Chalcolithic Populations

**DOI:** 10.1371/journal.pone.0105105

**Published:** 2014-08-12

**Authors:** Daniel Gómez-Sánchez, Iñigo Olalde, Federica Pierini, Laura Matas-Lalueza, Elena Gigli, Martina Lari, Sergi Civit, Marina Lozano, Josep Maria Vergès, David Caramelli, Oscar Ramírez, Carles Lalueza-Fox

**Affiliations:** 1 Institute of Evolutionary Biology (CSIC-Universitat Pompeu Fabra), Barcelona, Spain; 2 Laboratory of Anthropology, Department of Biology, University of Florence, Florence, Italy; 3 Department of Statistics, Faculty of Biology, University of Barcelona, Barcelona, Spain; 4 Institut Català de Paleoecologia Humana i Evolució Social, Tarragona, Spain; 5 Àrea de Prehistòria, Departament d’Història i Història de l’Art, Universitat Rovira i Virgili, Tarragona, Spain; University of York, United Kingdom

## Abstract

Previous mitochondrial DNA analyses on ancient European remains have suggested that the current distribution of haplogroup H was modeled by the expansion of the Bell Beaker culture (ca 4,500–4,050 years BP) out of Iberia during the Chalcolithic period. However, little is known on the genetic composition of contemporaneous Iberian populations that do not carry the archaeological tool kit defining this culture. Here we have retrieved mitochondrial DNA (mtDNA) sequences from 19 individuals from a Chalcolithic sample from El Mirador cave in Spain, dated to 4,760–4,200 years BP and we have analyzed the haplogroup composition in the context of modern and ancient populations. Regarding extant African, Asian and European populations, El Mirador shows affinities with Near Eastern groups. In different analyses with other ancient samples, El Mirador clusters with Middle and Late Neolithic populations from Germany, belonging to the Rössen, the Salzmünde and the Baalberge archaeological cultures but not with contemporaneous Bell Beakers. Our analyses support the existence of a common genetic signal between Western and Central Europe during the Middle and Late Neolithic and points to a heterogeneous genetic landscape among Chalcolithic groups.

## Introduction

The evolutionary history of the human settlement of Europe has been shaped by a complex pattern of migrations, driven by the appearance of new socio-economical strategies, cultural innovations and ecological changes [1]. This has created a specific genetic landscape that can be investigated from the analysis of current European populations [2]. However, modern-day genetic diversity provides only indirect evidence about the complex history of past populations.

Agriculture first developed in the Fertile Crescent of West Asia around 12–11,000 years ago, being afterwards developed independently over the next few thousand years in other regions of the planet [3]. The Neolithic shift to agriculture and the domestication of animals involved major demographic changes in the prehistoric populations, facilitating extensive human population growths and subsequent migrations [3].

Archaeological, anthropological and paleoecological evidence supports the existence of a major demographic change that took place with the arrival of the Neolithic around 9,000 years ago and the disappearance of the hunting and gathering strategy in the European continent after only few thousands of years [4].

However, the scale and nature of the interactions between local Mesolithic hunter-gatherers and incoming farmers has been a subject of much debate, opinions being divided between those who argue in favour of a population expansion from the Near East into Europe [5–9, amog others], and those who think that the process of ‘neolithisation’ was mainly a spread of ideas that led to the acculturation of the indigenous population [2], [4] with some potential regional variation [10]–[12].

In recent years, several ancient DNA studies have contributed to this debate by directly analysing ancient human skeletal remains, mainly Neolithic, and comparing their mtDNA composition to those of modern populations from the same area [12]–[22]. The interpretation of the results, obviously quite limited in sample size, has varied from clear genetic continuity from Neolithic to modern times, arrival of new mtDNA types from the Near East and possible hunter-gatherer acculturation in central and northern Europe. In addition, the vast majority of these studies have been restricted to the retrieval by polymerase chain reaction (PCR) of short fragments, predominantly from the hypervariable region-1 (HVR-1) of the mtDNA genome.

With the next-generation sequencing (NGS) technologies it has been possible to retrieve also prehistoric European genomic data. The first of these studies was the 7x coverage genome of the exceptionally well preserved Tyrolean Ice man, Ötzi, dated to about 5,300 years BP [23]. The second one was the partial genomic retrieval of one Neolithic farmer and four Neolithic hunter-gatherers from Scandinavia, dated between 4,400 and 5,300 years BP [24], [25], followed by the first retrieval of genomic data from two 7,000 years-old Mesolithic hunter-gatherers from La Braña-Arintero (León, Northwestern Spain) [26], [27]. These and future autosomal loci studies will enlarge the possibilities of selective and demographic analyses of the European prehistoric populations.

While most of efforts have focused on the Mesolithic-Neolithic transition, more recent periods remain relatively unknown [20], [21]. Nevertheless, it is likely that the first arrival of farmers was followed by a complex pattern of migrations and also regionalization episodes that can only be uncovered with the analysis of large numbers of individuals from different periods and geographical areas. At present the sequencing of nuclear genomes is a challenging task that will likely be restricted to a limited number of specimens, and thus the mtDNA can still provide valuable, additional information about these complex population processes.

To further investigate the transition between Late Neolithic to the Chalcolithic period, we have analyzed the mtDNA in a recently excavated population from El Mirador cave in Atapuerca, Spain. This site is contemporaneous to the Bell Beaker culture (BBC) but does not carry the diagnostic items of this culture that include the distinctive bell-shaped ceramics and weapons. In fact, the archaeological sites with Bell Beaker remains are very scarce in the Meseta Central of the Iberian Peninsula. It has been suggested [20] that the Bell Beaker culture represented a population movement from the Iberian Peninsula that could explain the genetic affinities between Central Europe’s Bell Beakers and present-day Iberians. Thus, the analysis of Iberian samples without the archaeological signature of the Bell Beaker culture such as El Mirador is of great interest to unravel the potential heterogeneity of the European Chalcolithic groups and its affinities with extant populations.

## Materials and Methods

**Figure 1:**
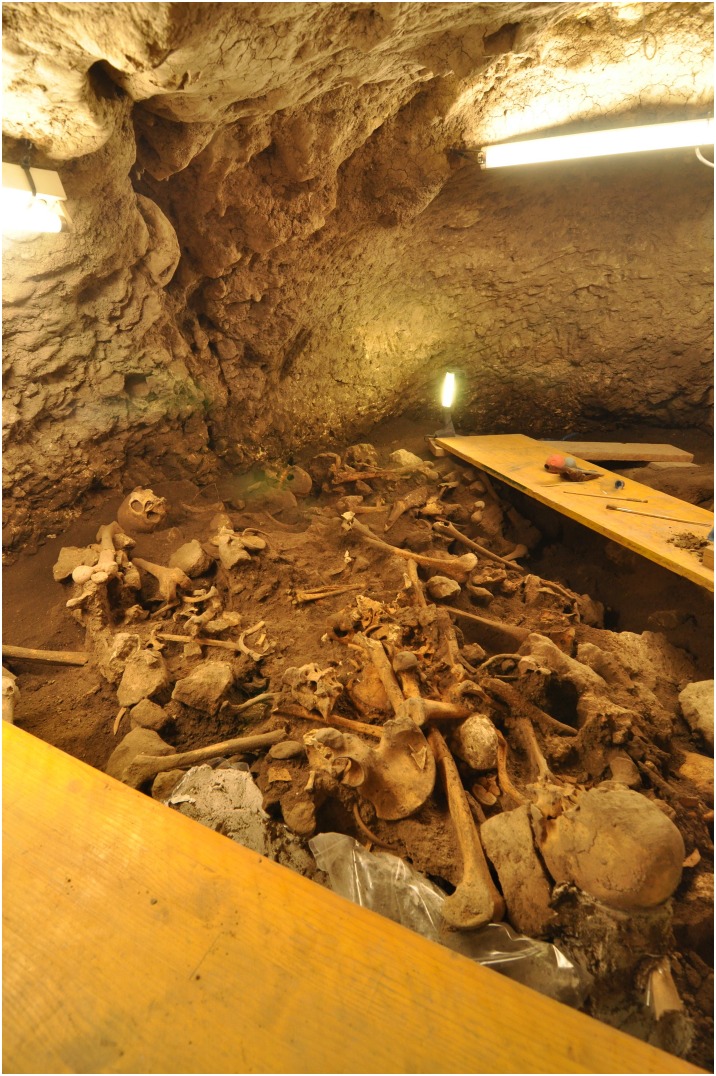
El Mirador site in Atapuerca (Spain).

The cave of El Mirador is located on the southern side of the Sierra de Atapuerca (Burgos, Spain), at an altitude of 1,033 meters above sea level (Fig. 1). The initial archaeological work was carried out in the 1970s by the Edelweiss Speleological Group. Subsequently an area of the cave covering about 20 m^2^ was affected by clandestine excavators. Later, in 1999, the archaeological fieldwork was resumed, and it is still continuing [28].

Two different assemblages of human remains were recovered from this site.

- The first human sample was recovered from a 6 m^2^ survey in an area unaffected by the clandestine workers. Six individuals dated to the Early Bronze Age (4,400–4,100 cal BP) were found. These human remains had been cannibalized and abandoned as rubbish in the Early Bronze Age, being buried later in the Middle or Late Bronze Age [29].
- After this first survey, in 2009 the fieldwork focused on a small cavity near the wall of the cave, where a collective burial was found. There were some individuals in anatomical position, but the most superficial remains had been disturbed and mixed by the earlier actions of the clandestine excavators. The human remains are associated with pottery without decoration, antler deer tines and fluvial shells. The fieldwork on this burial site is still in progress; however, a minimum number of 22 individuals have been recovered. All of these human remains belong to the Chalcolithic period and have been dated to 4,760–4,200 years cal. BP.

The Chalcolithic samples were left uncleaned until they were sampled for the genetic analysis, to prevent exogenous DNA contamination by handling. The samples analyzed are labelled Mirador 1, 2, 3, 4, 5, 6, 8, 9, 10, 11, 12, 13, 15, 16, 17, 18, 19, 20, 22, 23, 24, 25 and 26 (Table 1) and are deposited at the Institut Català de Paleoecologia Humana i Evolució Social (IPHES) in Tarragona (Spain). No permits were required for the described study.

**Table 1:** Mitochondrial DNA haplotype and haprogroup of El Mirador analysed samples.

Sample	Clones	HVR1 haplotype	Haplogroup
Mirador 1	34	294T 296T 324C	T2
Mirador 2	19	189C 223T 278T 352C	X
Mirador 3	27	CRS	H
Mirador 4	18	126C 294T 296T 324C	T2
Mirador 5	22	189C 223T 278T (358T)	X2
Mirador 6	17	189C 223T 278T	X2
Mirador 8 (1)	53	126C 294T 296T 304C	T2b
Mirador 9 (2)	23	189C 223T 278T	X2
Mirador 10	24	224C 256T 311C	K
Mirador 11	40	CRS	H
Mirador 12	32	224C 311C	K
Mirador 13	33	278T	H
Mirador 15	30	126C 294T 296T 304C	T2b
Mirador 16	18	093C 224T 311C	K
Mirador 18	17	CRS	H
Mirador 19	32	CRS	H
Mirador 20	38	069T 126C 193T 274A 278T	J1+16193/J2b
Mirador 22	30	224C 311C	K
Mirador 25	44	343G	U3
Mirador 26	36	069T 126C 145A 231C 261T	J2a1a

(1) Independently replicated in Florence laboratory.

(2) Mirador 5 and 9 correspond to two teeth from the same individual.

DNA was extracted from approximately 100 mg of dental root tips following the method described in detail in Lalueza-Fox et. al [30]. The sample was powdered and decalcified overnight with 10 ml 0.5 M EDTA pH 8.0 at 37°C; after centrifugation, the supernatant was removed and the remaining sample was incubated overnight at 50°C with 8.5 ml of water, 1 ml 5% SDS, 0.5 ml 1 M Tris-HCl pH 8.0 and 50 µl of 1 mg/ml Proteinase K. After incubation, the digest was extracted three times, first with phenol, second with phenol-chloroform and third with chloroform, and the aqueous phase was concentrated by dialysis centrifugation using Amicon Centrifugal Filter Devices (Amicon, Millipore). Subsequent purification with silica yielded a final 30 µl extract.

Extraction procedures were performed in an isolated pre-PCR area, adopting the standard precautions of ancient DNA studies [31]–[33]. Multiple extraction and amplification negative controls to monitor for contamination in the reagents were added to each PCR reaction. No amplification products were obtained in these blank PCR controls along the study.

Besides standard precautions in ancient DNA outlined, we followed several additional strategies to support the authenticity of our results. 1) we retrieved endogenous DNA sequences from an animal bone (wolf) from the same site, to ensure that the cave environment was favourable indeed to DNA preservation, 2) we retrieved the same mtDNA haplotype from two teeth belonging to the same individual, 3) we replicated mtDNA sequences of an individual that shares the same mtDNA haplotype with two of the molecular researchers in an independent laboratory (Florence) after sending an additional sample directly from the archaeological institution, and 4) we repeated the amplification and sequencing of potentially rare haplotypes.

In Barcelona, the mtDNA hypervariable region 1 (HVR1) was amplified by polymerase chain reaction (PCR) in two overlapping fragments (L16055-H16218 and L16185-H16378 primers), following a two-steps protocol (27 and 33 cycles of PCR, respectively), in which primers are limited in the first PCR step to avoid the generation of unspecific primer-primer artifacts [30]. In Florence the HVR1 was retrieved in three overlapping fragments (L16995-H16132, L16107-H16247 and L16222-H16327).

In both laboratories, PCR products were subsequently visualized under UV lights in a low-melting point agarose gel. The appropriate bands were excised from the gel, melted at 65° for 20 min, and purified with a silica-based method. Subsequently, the amplification products were cloned in bacteria (TOPO-TA cloning kit, Invitrogen). Resulting white colonies were picked up, amplified with M13 universal primers and sequenced in an ABI3730 capillary sequencer (Applied Biosystems).

The haplogroup frequencies data were classified as described in Brandt et al. [21] to allow comparison with their results and analysed with R (version 3.1.0). Principal component analyses (PCA) were performed with *prcomp* function using mtDNA haplogroup diversity (Table S1, S2 and S3) to determine population affinities between El Mirador and prehistoric/present-day European populations. Manhattan distances and 10,000 bootstrap replicates were used to perform hierarchical Ward clustering with *pvclust* function [34].

## Results and Discussion

From the analysis of 757 clone sequences (Figure S1), we have obtained the mtDNA HVR1 in 19 individuals, accounting for different mtDNA haplogroups and sub-haplogroups previously described in Europe (Table 1). This success rate is remarkably high and could be attributed to the thermal stability favoured by the cave environment, as opposed for instance to open field sites. Other sites at Atapuerca, including Sima de los Huesos, dated to about 400,000 years ago, have yielded endogenous DNA [35]. El Mirador consensus sequences are deposited at GenBank under accession numbers KJ1866158–KJ1866171, KJ1866173–KJ1866176 and KJ1866179–KJ1866180. To differentiate between endogenous substitutions and potential *postmortem* damage or background contamination (the latter most frequently will emerge as CRS sequences, the most common haplotype in Europeans today) we have replicated at least twice most of the mtDNA fragments, as recommended in Hofreiter et al. [32]. Three samples (Mir17, Mir23 and Mir24) yielded mixed haplotypes in different PCRs and were discarded for subsequent analyses.

Despite the recent retrieval of partial or complete Mesolithic and Neolithic genomes [24]–[27], the analysis of the mtDNA lineages remains the most widely used marker for reconstructing the human matrilineal population affinities. Recent analyses of partial and complete ancient mtDNA genomes have revealed different events associated to the distribution and diversity of these lineages in modern Europeans [20], [21]. The analysis of the mtDNA composition along time detected a previously unrecognised major genetic transition between the Early Neolithic and posterior Middle and Late Neolithic periods [20]. While many H haplogroup (the most common in modern Europeans, with frequencies around 40%) lineages were established by the Middle Neolithic period, a subsequent migration movement in the Late Neolithic associated to the Bell Beaker culture added further genetic complexity to the present-day populations. The genetic signature of H haplogroups increased up to 48.3% during the Bell Beaker period with respect to previous European cultures, suggesting a population expansion from Iberia to Central Europe [21].

However, another recent study based on the analysis of 629,443 single nucleotide polymorphisms (SNPs) from 934 individuals belonging to 53 human populations has uncovered a previously unrecognized signature of Northern European genes into the Iberian Peninsula [36]. Based on the length distribution of the linkage-disequilibrium blocks, it has been possible to date this admixture event to about 4,000 years BP, a figure roughly coincident with the spread of the Bell Beaker culture [36]. They interpret this signal as the result of a reverse migration from central Europe into Iberia after an initial Bell Beaker culture expansion from Iberia. This has been previously hypothesized from archaeological data [37] but so far has not been observed with ancient genetic data due to the current lack of genetic information from Iberian Bell Beaker groups.

**Figure 2:**
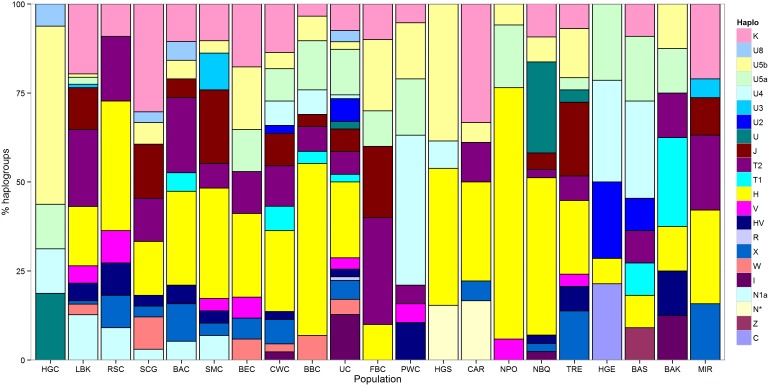
Mitochondrial DNA haplogroup frequency for 21 ancient European samples. This study: El Mirador (MIR). Published prehistoric cultures [21]: Hunter-gatherer central (HGC), Linear Pottery culture (LBK), Rössen culture (RSC), Schöningen group (SCG), Baalberge culture (BAC), Salzmünde culture (SMC), Bernburg culture (BEC), Corded Ware culture (CWC), Bell Beaker culture (BBC), Unetice culture (UC), Funnel Beaker culture (FBC), Pitted Ware culture (PWC), Hunter-Gatherer south (HGS), (Epi) Cardial (CAR), Neolithic Portugal (NPO), Neolithic Basque Country and Navarre (NBQ), Treilles culture (TRE), Hunter-gatherer east (HGE), Bronze Age Siberia (BAS), Bronze Age Kazakhstan (BAK).

**Figure 3:**
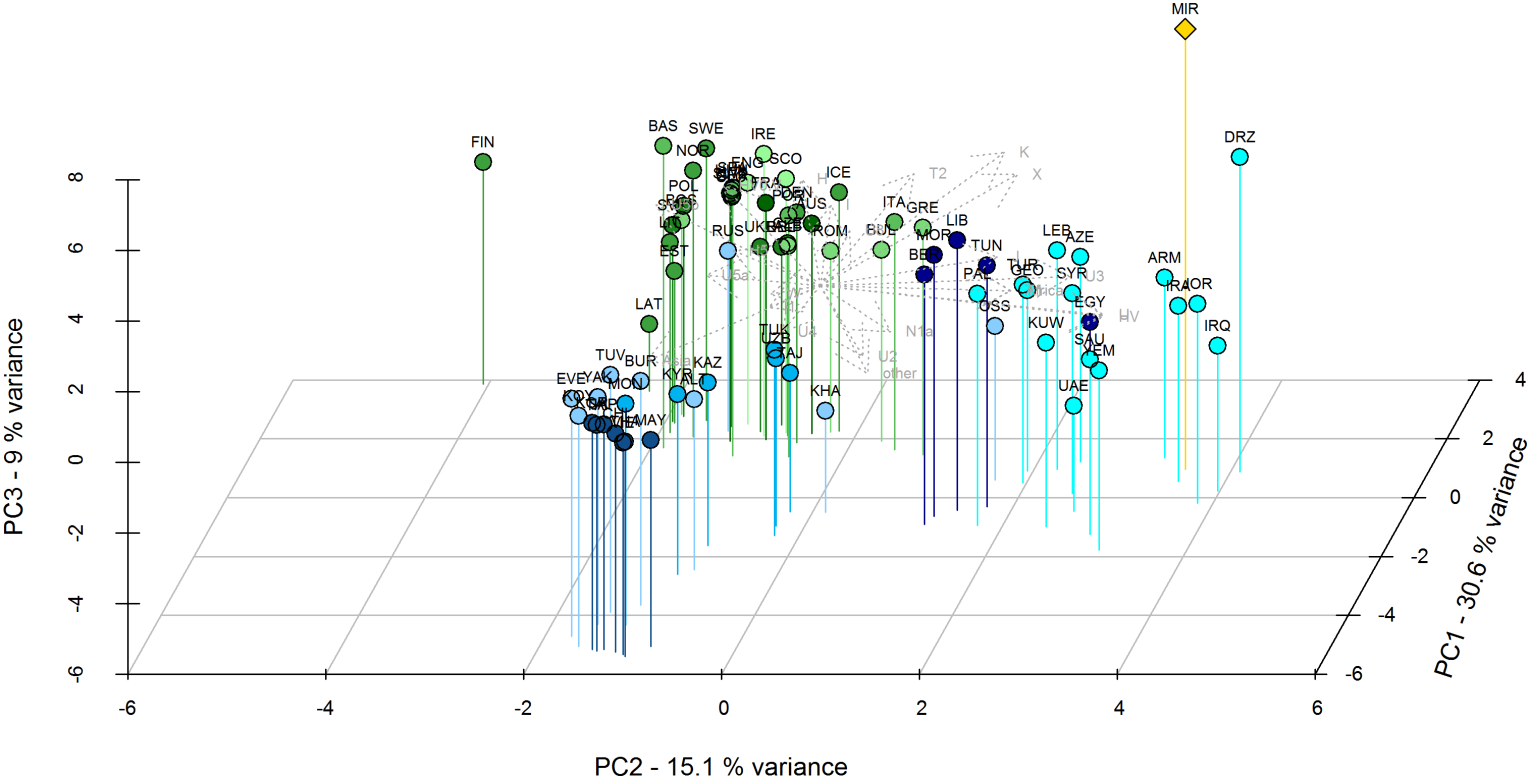
Principal component analysis of El Mirador and present-day African, Asian and European populations. Principal component analysis (PCA) based on mitochondrial haplogroup frequencies of El Mirador (MIR, yellow) and published present-day populations [21]: African/Asian (in blue, from darker to lighter North Africa, Southwest Asia, Central Asia, North Asia and Southwest Asia) and European (in green, from darker to lighter, Central, East, North, South, Southwest and West). For further information about populations, abbreviation and frequencies, see Table S1. The first three principal components display the 54.5% of the total variance.

The mtDNA composition of El Mirador is quite unique, and different to that found in other contemporaneous Bell Beaker populations and to present-day Iberians (Figure 2 and 3). This Chalcolithic population displays different mtDNA haplogroups that are currently present at higher frequency in the Near East populations than in continental Europe (e.g., X2, K, T2b); this could explain the clustering of El Mirador with Near Eastern populations in the PCA of modern populations (Figure 3). The Near East signature found here could correspond to the major genetic transition detected by Brandt et al. [21] between the Early and Late Neolithic and could indicate a subsequent migratory movement into Europe from the Near East, maybe associated to cultural transitions such as Megalithism.

**Figure 4:**
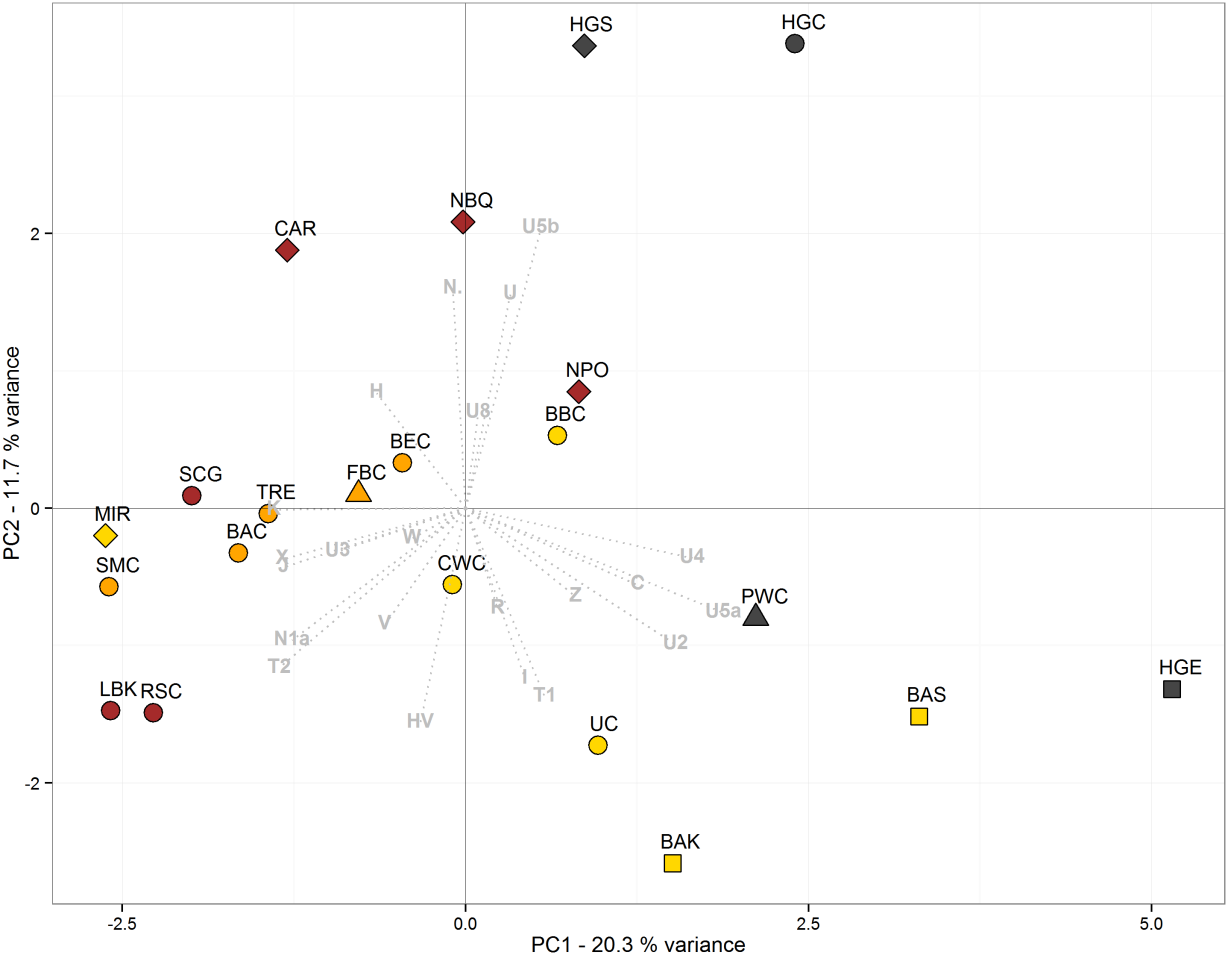
Principal component analysis of El Mirador and other prehistoric cultures. Principal component analysis (PCA) based on mitochondrial haplogroup frequencies of El Mirador (MIR) and 20 published prehistoric cultures [21]: Hunter-gatherer central (HGC), Linear Pottery culture (LBK), Rössen culture (RSC), Schöningen group (SCG), Baalberge culture (BAC), Salzmünde culture (SMC), Bernburg culture (BEC), Corded Ware culture (CWC), Bell Beaker culture (BBC), Unetice culture (UC), Funnel Beaker culture (FBC), Pitted Ware culture (PWC), Hunter-Gatherer south (HGS), (Epi) Cardial (CAR), Neolithic Portugal (NPO), Neolithic Basque Country and Navarre (NBQ), Treilles culture (TRE), Hunter-gatherer east (HGE), Bronze Age Siberia (BAS), Bronze Age Kazakhstan (BAK). Symbols indicate populations from Central Europe (circles), southern Scandinavia (triangles), the Iberian Peninsula (diamonds), and East Europe/Asia (squares). Colour indicates hunter-gatherer (grey), Early Neolithic (brown), Middle Neolithic (orange), and Late Neolithic/EBA (yellow) samples. For further information, see Table S2. The first two components display the 32.1% of the total variance.

**Figure 5:**
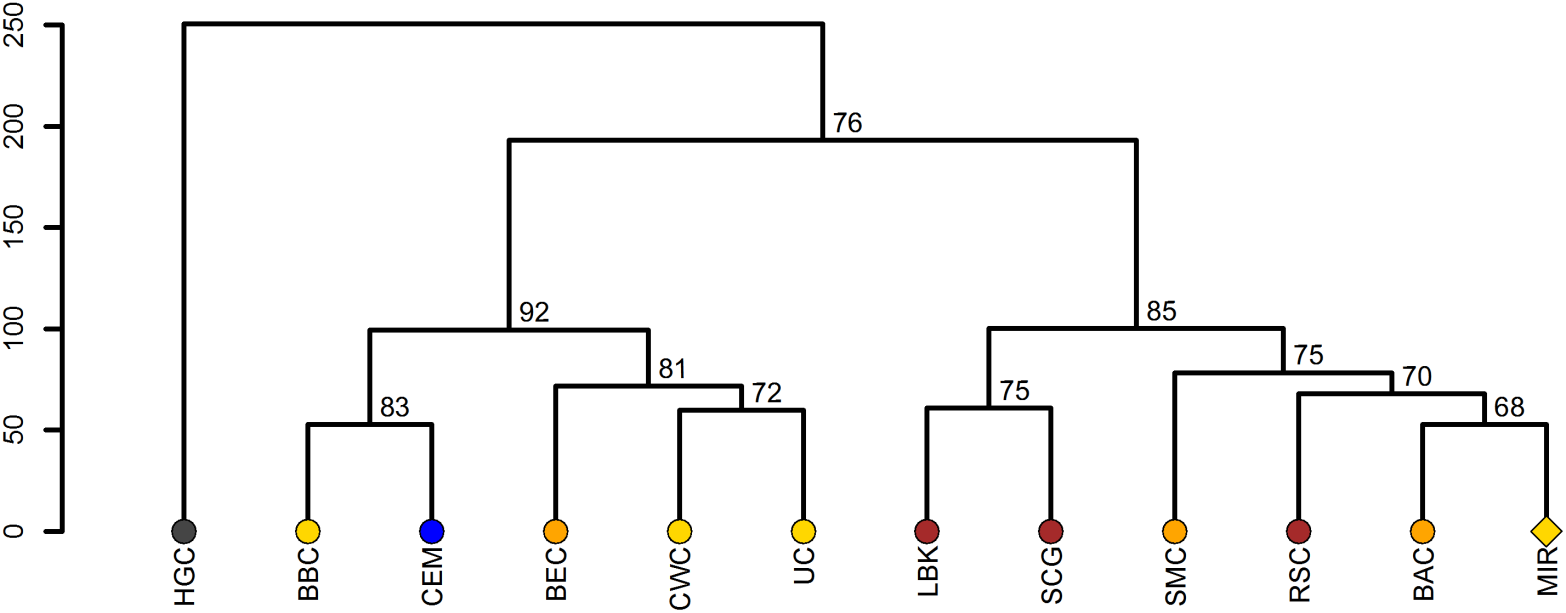
Hierarchical Ward clustering of El Mirador along with modern and prehistoric samples from Central Europe. Haplogroup frequencies of El Mirador (MIR), hunter-gatherer central (HGC), and nine Mittelelbe-Saale (Saxony-Anhalt region in Germany) cultures [21] have been used to generate a hierarchical Ward clustering. Linear Pottery culture (LBK), Rössen culture (RSC), Schöningen group (SCG), Baalberge culture (BAC), Salzmünde culture (SMC), Bernburg culture (BEC), Corded Ware culture (CWC), Bell Beaker culture (BBC), Unetice culture (UC), present-day Central European metapopulation (CEM) (N = 500). For further information, see Table S3. The *p-values* of the clusters are given in percentage of reproduced clusters based on 10,000 bootstrap replicates.

The clustering of the El Mirador sample with a Salzmünde culture (5,400-5,100/5,025 years BP) population from Germany and the roughly contemporaneous Treilles culture population from France in the PCA of ancient populations (Figure 4 and Figure S2) as well as with the previous Rössen and Baalberge cultures from Germany in the hierarchical Ward clustering (Figure 5) supports the existence of a common genetic signal among Western and Central Europe during the Middle and Late Neolithic. In this context, El Mirador mitochondrial composition may correspond to a previous genetic substratum with a substantial contribution of lineages from the Near East that was not influenced by the expansion of Iberian Bell Beakers, despite being in the same range. Under a chronological perspective (Figure S3) El Mirador supports the continuity of the previous Middle Neolithic genetic composition into the Chalcolithic, at least in non-Bell Beaker groups.

To explore the genetic signal of these population affinities, we have estimated the correlation coefficient between haplogroups and PCA dimensions and performed a significance test (Table 2). K, T2, J, X and also N1a are the haplogroups that clearly influence the separation of the Early/Middle Neolithic cultures from Germany -including also El Mirador- along the first dimension, as opposed to U4 and U5a haplogroups. U and U5b explain the separation of the hunter-gatherers groups along the second dimension.

**Table 2:** Correlation coefficients and *p-values* of the mtDNA haplogroups significantly correlated to the two principal axes in the PCA from the ancient populations.

Component	Haplogroup	Correlation	*p-value*
PC1	K	−0.621	2.65E-03
	T2	−0.604	3.76E-03
	J	−0.592	4.67E-03
	X	−0.589	4.99E-03
	N1a	−0.561	8.17E-03
	C	0.558	8.64E-03
	U2	0.682	6.66E-04
	U4	0.735	1.49E-04
	U5a	0.836	2.36E-06
PC2	HV	−0.526	0.0142
	T1	−0.463	0.0347
	U	0.524	0.0147
	N	0.538	0.0119
	U5b	0.690	0.0006

In none of the analyses El Mirador sample shows close genetic affinities with a contemporaneous Bell Beaker population of 29 specimens gathered from three sites in Germany. The Bell Beaker mtDNA signal is characterized by high frequencies (around 50%) of H haplogroup that in El Mirador only reaches 26%. This heterogeneity in the genetic composition of geographically close populations adds further complexity to future reconstructions of these ancient expansions and correlates with the existence of contemporaneous groups with and without the typical Bell Beaker burial kit.

The generation of complete genomes with the new sequencing technologies will likely contribute to provide a more detailed picture of the complex population movements and affinities along the European prehistory and would eventually explain the dynamics of pan-European Chalcolithic migrations.

## Supporting Information

Figure S1 **mtDNA clone sequences of the El Mirador samples.** Samples with potentially conflicting haplotypes or with substitutions that could be attributed to *postmortem* damage were repeatedly amplified to generate a consensus sequence. Some CRS clones attributable to background contamination have been suppressed for clarity.
(PDF)

Figure S2 **Boxplot grouped by geography of El Mirador principal components and other prehistoric cultures.** PC1 and PC2 show the differentiation between geographical distributions of ancient cultures. El Mirador population (in yellow) shows affinities with prehistoric Central European cultures despite its location in Spain.
(TIFF)

Figure S3 **Boxplot grouped by chronology of El Mirador principal components and other prehistoric cultures.** PC1 shows the differentiation between hunter-gatherer, Early/Middle Neolithic and Late Neolithic/EBA, whereas PC2 gives information for all the periods. El Mirador population (yellow) have traits of the Early/Middle Neolithic period in these components despite its Chalcolithic attribution.
(TIFF)

Table S1 **Details and haplogroup frequencies of El Mirador and present-day populations used in principal component analysis.** Relative haplogroup frequencies of El Mirador and previously published populations [21] were used for principal component analysis. Haplogroups were classified into 23 groups.
(XLSX)

Table S2 **Details and haplogroup frequencies of El Mirador and prehistoric cultures used in principal component analysis.** Relative haplogroup frequencies of El Mirador and previously published cultures [21] were used for principal component analysis. Haplogroups were classified into 22 groups.
(XLSX)

Table S3 **Details and haplogroup frequencies of El Mirador, Central European metapopulation and prehistoric cultures used in Ward’s hierarchical clustering.** Relative haplogroup frequencies of El Mirador and previously published populations [21] were used for hierarchical Ward clustering. Haplogroups were classified into 20 groups.
(XLSX)
