# Supplementary material for: Mitochondrial DNA from El Mirador Cave (Atapuerca, Spain) Reveals the Heterogeneity of Chalcolithic Populations
Source: PLoS One. 2014 Aug 12;9(8):e105105. doi: 10.1371/journal.pone.0105105 (PMC4130614; doi:10.1371/journal.pone.0105105)
Supplement: Figure S1 — mtDNA clone sequences of the El Mirador samples. Samples with potentially conflicting haplotypes or with substitutions that could be attributed to postmortem damage were repeatedly amplified to generate a consensus sequence. Some CRS clones attributable to background contamination have been suppressed for clarity. (PDF) [file pone.0105105.s001.pdf]

Mirador1 294 296 324 T2*

```
               111111111111111111111111111111111111111111111111111111111111111111111111111111111111111111111111111111111111111111111111111111111111111111111111111111111111111111111111111111111111111111111111111111111111111111111111111111111111111111111111111111111111111111111111111111111111111111111111111111111111111111111111111111111111111111111111111111111111111111111111111111
               666666666666666666666666666666666666666666666666666666666666666666666666666666666666666666666666666666666666666666666666666666666666666666666666666666666666666666666666666666666666666666666666666666666666666666666666666666666666666666666666666666666666666666666666666666666666666666666666666666666666666666666666666666666666666666666666666666666666666666666666666666
               000000000000000000000000000000000000000000000000000000000000000000000000000001111111111111111111111111111111111111111111111111111111111111111111111111111111111111111111111111111122222222222222222222222222222222222222222222222222222222222222222222222222222222222222222222222222223333333333333333333333333333333333333333333333333333333333333333333333333333333333333333333333334
               222222233333333334444444444555555555566666666667777777777888888888899999999990000000000111111111122222222223333333333444444444455555555556666666666777777777788888888889999999999000000000011111111112222222222333333333344444444445555555555666666666677777777778888888888999999999900000000001111111111222222222233333333334444444444555555555566666666667777777777888888888899999999990
               345678901234567890123456789012345678901234567890123456789012345678901234567890123456789012345678901234567890123456789012345678901234567890123456789012345678901234567890123456789012345678901234567890123456789012345678901234567890123456789012345678901234567890123456789012345678901234567890123456789012345678901234567890123456789012345678901234567890123456789012345678901234567890 PCR1
               GTTCTTTCATGGGGAAGCAGATTTGGGTACCACCCAAGTATTGACTCACCCATCAACAACCGCTATGTATTTCGTACATTACTGCCAGCCACCATGAATATTGTACGGTACCATAAATACTTGACCACCTGTAGTACATAAAAACCCAATCCACATCAAAACCCCCTCCCCATGCTTACAAGCAAGTACAGCAATCAACCCTCAACTATCACACATCAACTGCAACTCCAAAGCCACCCCTCACCCACTAGGATACCAACAAACCTACCCACCCTTAACAGTACATAGTACATAAAGCCATTTACCGTACATAGCACATTACAGTCAAATCCCTTCTCGTCCCCATGGATGACCCCCCTCAGATAGGGGTCCCTTGAC
Clone 1 PCR1                              L16055..............C.......................................................C...........................................................................................H16218
Clone 2 PCR1                                    ...............................................................................................................................C.....C............................
Clone 3 PCR1                                    ..................................................................................................................................................................
Clone 4 PCR1                                    ....................................................................................................................................T.............................
Clone 5 PCR1                                    ..................................................................................................................................................................
Clone 6 PCR1                                    ..................................................................................................................................................................
Clone 7 PCR1                                    .......................................N............................................................................................T...........T...A.............
Clone 1 PCR2                                                                                                                                                                    L16185..........................................T.................................................................T.T...........................C.....................................................H16378
Clone 2 PCR2                                                                                                                                                                          .............................................................................................................................C............C.....................................................
Clone 3 PCR2                                                                                                                                                                          .............................................................................................................................C..................................................................
Clone 4 PCR2                                                                                                                                                                          .........................................G..................................................................T.T...........................C.................................T...................
Clone 5 PCR2                                                                                                                                                                          ..............................................................................................................T.............................................................T...................
Clone 1 PCR3                                                                                                                                                                    L16185............................................................................................................T.T...........................C.....................................................H16378
Clone 2 PCR3                                                                                                                                                                          ............................................................................................................T.T...........................C.....................................................
Clone 3 PCR3                                                                                                                                                                          ............................................................................................................T.T...........................C.....................................................
Clone 4 PCR3                                                                                                                                                                          ............C...............................................................................................T.T...........................C.....................................................
Clone 5 PCR3                                                                                                                                                                          ............................................................................................................T.T...........................C.....................................................
Clone 6 PCR3                                                                                                                                                                          ............................................................................................................T.T...........................C.....................................................
Clone 7 PCR3                                                                                                                                                                          ............................................................................................................T.T...........................C.....................................................
Clone 8 PCR3                                                                                                                                                                          ............................................................................................................T.T...........................C.....................................................
Clone 10 PCR3                                                                                                                                                                         ............................................................................................................T.T...........................C.....................................................
Clone 11 PCR3                                                                                                                                                                         ............................................................................................................T.T...........................C.....................................................
Clone 12 PCR3                                                                                                                                                                         ............................................................................................................T.T...........................C.....................................................
Clone 13 PCR3                                                                                                                                                                         ..............................................................................T.............................T.T...........................C.....................................................
Clone 14 PCR3                                                                                                                                                                         ............................................................................................................T.T...........................C.....................................................
Clone 15 PCR3                                                                                                                                                                         ............................................................................................................T.T.................................................................................
Clone 16 PCR3                                                                                                                                                                         ............................................................................................................T...................................................................................
Clone 17 PCR3                                                                                                                                                                         ............................................................................................................T.T...........................C.....................................................
Clone 18 PCR3                                                                                                                                                                         ............................................................................................................T.T...........................C.....................................................
Clone 19 PCR3                                                                                                                                                                         ...C................................................................................T...A.......................................................................................................
Clone 20 PCR3                                                                                                                                                                         ...........G...........................................GG...................................................T.T...........................C.....................................................
```

Mirador2 189C 223T 278T 352C X*

```
               111111111111111111111111111111111111111111111111111111111111111111111111111111111111111111111111111111111111111111111111111111111111111111111111111111111111111111111111111111111111111111111111111111111111111111111111111111111111111111111111111111111111111111111111111111111111111111111111111111111111111111111111111111111111111111111111111111111111111111111111111111
               666666666666666666666666666666666666666666666666666666666666666666666666666666666666666666666666666666666666666666666666666666666666666666666666666666666666666666666666666666666666666666666666666666666666666666666666666666666666666666666666666666666666666666666666666666666666666666666666666666666666666666666666666666666666666666666666666666666666666666666666666666
               000000000000000000000000000000000000000000000000000000000000000000000000000001111111111111111111111111111111111111111111111111111111111111111111111111111111111111111111111111111122222222222222222222222222222222222222222222222222222222222222222222222222222222222222222222222222223333333333333333333333333333333333333333333333333333333333333333333333333333333333333333333333334
               222222233333333334444444444555555555566666666667777777777888888888899999999990000000000111111111122222222223333333333444444444455555555556666666666777777777788888888889999999999000000000011111111112222222222333333333344444444445555555555666666666677777777778888888888999999999900000000001111111111222222222233333333334444444444555555555566666666667777777777888888888899999999990
               345678901234567890123456789012345678901234567890123456789012345678901234567890123456789012345678901234567890123456789012345678901234567890123456789012345678901234567890123456789012345678901234567890123456789012345678901234567890123456789012345678901234567890123456789012345678901234567890123456789012345678901234567890123456789012345678901234567890123456789012345678901234567890 PCR1
               GTTCTTTCATGGGGAAGCAGATTTGGGTACCACCCAAGTATTGACTCACCCATCAACAACCGCTATGTATTTCGTACATTACTGCCAGCCACCATGAATATTGTACGGTACCATAAATACTTGACCACCTGTAGTACATAAAAACCCAATCCACATCAAAACCCCCTCCCCATGCTTACAAGCAAGTACAGCAATCAACCCTCAACTATCACACATCAACTGCAACTCCAAAGCCACCCCTCACCCACTAGGATACCAACAAACCTACCCACCCTTAACAGTACATAGTACATAAAGCCATTTACCGTACATAGCACATTACAGTCAAATCCCTTCTCGTCCCCATGGATGACCCCCCTCAGATAGGGGTCCCTTGAC
Clone 1 PCR1                              L16055......................................C.............................................................................................TC............................H16218
Clone 2 PCR1                                    .....................................................................................................................................C.......T....................
Clone 3 PCR1                                    .......................................N..................T.............................................................T............C............................
Clone 4 PCR1                                    .......................................N.............................................................................................CTTT.........................
Clone 5 PCR1                                    .......................................N.............................................................................................C.......T....................
Clone 6 PCR1                                                                                                                                                                    L16185...C.................................T......................................................T.........................................................................C.........................H16378
Clone 1 PCR2                                                                                                                                                                          ...C.................................T......................................................T.........................................................................C.....T.T.................
Clone 2 PCR2                                                                                                                                                                          ...C.................................T......................................................T.........................................................................C.........................
Clone 3 PCR2                                                                                                                                                                          ............................................................................................T.........................................................................C.........................
Clone 4 PCR2                                                                                                                                                                          ...C........................................................................................T...........T.T.....................................................................................
Clone 5 PCR2                                                                                                                                                                          ...A...........................................................C........................A...T.....G....C...........T.........C...........................................T............C.........
Clone 1 PCR3                                                                                                                                                                    L16185...C.................................T......................................................T.........................................................................C.........................H16378
Clone 2 PCR3                                                                                                                                                                          ...C.................................T......................................................T.........................................................................C.........................
Clone 3 PCR3                                                                                                                                                                          ...C.................................T......................................................T...................................................................................................
Clone 4 PCR3                                                                                                                                                                          ...C.................................T...................................................T..T.........................................................................C.........................
Clone 5 PCR3                                                                                                                                                                          ...C.................................T......................................................T...................................................................................................
```

Mirador3 CRS

```
               111111111111111111111111111111111111111111111111111111111111111111111111111111111111111111111111111111111111111111111111111111111111111111111111111111111111111111111111111111111111111111111111111111111111111111111111111111111111111111111111111111111111111111111111111111111111111111111111111111111111111111111111111111111111111111111111111111111111111111111111111111
               666666666666666666666666666666666666666666666666666666666666666666666666666666666666666666666666666666666666666666666666666666666666666666666666666666666666666666666666666666666666666666666666666666666666666666666666666666666666666666666666666666666666666666666666666666666666666666666666666666666666666666666666666666666666666666666666666666666666666666666666666666
               000000000000000000000000000000000000000000000000000000000000000000000000000001111111111111111111111111111111111111111111111111111111111111111111111111111111111111111111111111111122222222222222222222222222222222222222222222222222222222222222222222222222222222222222222222222222223333333333333333333333333333333333333333333333333333333333333333333333333333333333333333333333334
               222222233333333334444444444555555555566666666667777777777888888888899999999990000000000111111111122222222223333333333444444444455555555556666666666777777777788888888889999999999000000000011111111112222222222333333333344444444445555555555666666666677777777778888888888999999999900000000001111111111222222222233333333334444444444555555555566666666667777777777888888888899999999990
               345678901234567890123456789012345678901234567890123456789012345678901234567890123456789012345678901234567890123456789012345678901234567890123456789012345678901234567890123456789012345678901234567890123456789012345678901234567890123456789012345678901234567890123456789012345678901234567890123456789012345678901234567890123456789012345678901234567890123456789012345678901234567890 PCR1
               GTTCTTTCATGGGGAAGCAGATTTGGGTACCACCCAAGTATTGACTCACCCATCAACAACCGCTATGTATTTCGTACATTACTGCCAGCCACCATGAATATTGTACGGTACCATAAATACTTGACCACCTGTAGTACATAAAAACCCAATCCACATCAAAACCCCCTCCCCATGCTTACAAGCAAGTACAGCAATCAACCCTCAACTATCACACATCAACTGCAACTCCAAAGCCACCCCTCACCCACTAGGATACCAACAAACCTACCCACCCTTAACAGTACATAGTACATAAAGCCATTTACCGTACATAGCACATTACAGTCAAATCCCTTCTCGTCCCCATGGATGACCCCCCTCAGATAGGGGTCCCTTGAC
Clone 1 PCR1                              L16055..............................................................A..................................A................................................................H16218
Clone 2 PCR1                                    ..................................................................................................................................T...............................
Clone 3 PCR1                                    .............T....................................................................................................................................................
Clone 4 PCR1                                    .............................................................................................................................................A....................
Clone 5 PCR1                                    ...........................T......................................................................................................................................
Clone 6 PCR1                                    ..................................................................................................................................................................
Clone 7 PCR1                                    ..................G...............................................................................................................................................
Clone 8 PCR1                                    ..................................................................................................................................................................
Clone 9 PCR1                                    ..................................................................................................................................................................
Clone 10 PCR1                                   .................T.....T..TT......................................................................................................................................
Clone 11 PCR1                                   ...................................................TT.....................................A.......................................................................
Clone 1 PCR2                                                                                                                                                                    L16185.............................................................................................................................C..................................................................H16378
Clone 2 PCR2                                                                                                                                                                          .T...T.....T...............................................................TT.......T.......T...................................................................................................
Clone 3 PCR2                                                                                                                                                                          ...................A............................................................TT..T...........................................................................................................
Clone 4 PCR2                                                                                                                                                                          ................................................................................................................................................................................................
Clone 5 PCR2                                                                                                                                                                          ................................................................................................................................................................................................
Clone 6 PCR2                                                                                                                                                                          ................................................................................................................................................................................................
Clone 7 PCR2                                                                                                                                                                          ................................................................................................................................................................................................
Clone 8 PCR2                                                                                                                                                                          ................................................................................................................................................................................................
Clone 9 PCR2                                                                                                                                                                          ................................................................................................................................................................................................
Clone 10 PCR2                                                                                                                                                                         ................................................................................................................................................................................................
Clone 11 PCR2                                                                                                                                                                         ................................................................................................................................................................................................
Clone 12 PCR2                                                                                                                                                                         ................................................................................................................................................................................................
Clone 13 PCR2                                                                                                                                                                         ................................................................................................................................................................................................
Clone 14 PCR2                                                                                                                                                                         ................................................................................................................................................................................................
Clone 15 PCR2                                                                                                                                                                         ................................................................................................................................................................................................
Clone 16 PCR2                                                                                                                                                                         ................................................................................................................................................................................................
```

Mirador4 126 294 296 324 T2a1b*

```
               111111111111111111111111111111111111111111111111111111111111111111111111111111111111111111111111111111111111111111111111111111111111111111111111111111111111111111111111111111111111111111111111111111111111111111111111111111111111111111111111111111111111111111111111111111111111111111111111111111111111111111111111111111111111111111111111111111111111111111111111111111
               666666666666666666666666666666666666666666666666666666666666666666666666666666666666666666666666666666666666666666666666666666666666666666666666666666666666666666666666666666666666666666666666666666666666666666666666666666666666666666666666666666666666666666666666666666666666666666666666666666666666666666666666666666666666666666666666666666666666666666666666666666
               000000000000000000000000000000000000000000000000000000000000000000000000000001111111111111111111111111111111111111111111111111111111111111111111111111111111111111111111111111111122222222222222222222222222222222222222222222222222222222222222222222222222222222222222222222222222223333333333333333333333333333333333333333333333333333333333333333333333333333333333333333333333334
               222222233333333334444444444555555555566666666667777777777888888888899999999990000000000111111111122222222223333333333444444444455555555556666666666777777777788888888889999999999000000000011111111112222222222333333333344444444445555555555666666666677777777778888888888999999999900000000001111111111222222222233333333334444444444555555555566666666667777777777888888888899999999990
               345678901234567890123456789012345678901234567890123456789012345678901234567890123456789012345678901234567890123456789012345678901234567890123456789012345678901234567890123456789012345678901234567890123456789012345678901234567890123456789012345678901234567890123456789012345678901234567890123456789012345678901234567890123456789012345678901234567890123456789012345678901234567890 PCR1
               GTTCTTTCATGGGGAAGCAGATTTGGGTACCACCCAAGTATTGACTCACCCATCAACAACCGCTATGTATTTCGTACATTACTGCCAGCCACCATGAATATTGTACGGTACCATAAATACTTGACCACCTGTAGTACATAAAAACCCAATCCACATCAAAACCCCCTCCCCATGCTTACAAGCAAGTACAGCAATCAACCCTCAACTATCACACATCAACTGCAACTCCAAAGCCACCCCTCACCCACTAGGATACCAACAAACCTACCCACCCTTAACAGTACATAGTACATAAAGCCATTTACCGTACATAGCACATTACAGTCAAATCCCTTCTCGTCCCCATGGATGACCCCCCTCAGATAGGGGTCCCTTGAC
Clone 1 PCR1                              L16055......................................................................C...........................................................................................H16218
Clone 2 PCR1                                    ......................................................................C...........................................................................................
Clone 3 PCR1                                    ......................................................................C...........................................................................................
Clone 4 PCR1                                    .......................................T..............................C...........................................................................................
Clone 5 PCR1                                    ......................................................................C...........................................................................................
Clone 6 PCR1                                    .......................................T..............................C...........................................................................................
Clone 7 PCR1                                    ......................................................................C...........................................................................................
Clone 1 PCR2                                                                                                                                                                    L16185............................................................................................................T.T...........................C...................................T.................H16378
Clone 2 PCR2                                                                                                                                                                          ............................................................................................................T.T...........................C..................................T..................
Clone 3 PCR2                                                                                                                                                                          ............................................................................................................T.T...........................C.....................................................
Clone 4 PCR2                                                                                                                                                                          ............................................................................................................T.T...........................C.....................................................
Clone 5 PCR2                                                                                                                                                                          ............................................................................................................T.T...........................C.....................................................
Clone 6 PCR2                                                                                                                                                                          ...................................................G........................................................T.T...........................C.....................................................
Clone 7 PCR2                                                                                                                                                                          ............................................................................................................T.T...........................C.....................................................
Clone 8 PCR2                                                                                                                                                                          ............................................................................................................T.T...........................C.....................................................
Clone 9 PCR2                                                                                                                                                                          ............................................................................................................T.T...........................C.............................................A.......
Clone 10 PCR2                                                                                                                                                                         ............................................................................................................T.T...........................C.....................................................
```

```
Clone 11 PCR2

Mirador5 189 223 278 (358) X*
               111111111111111111111111111111111111111111111111111111111111111111111111111111111111111111111111111111111111111111111111111111111111111111111111111111111111111111111111111111111111111111111111111111111111111111111111111111111111111111111111111111111111111111111111111111111111111111111111111111111111111111111111111111111111111111111111111111111111111111111111111111111111111111
               666666666666666666666666666666666666666666666666666666666666666666666666666666666666666666666666666666666666666666666666666666666666666666666666666666666666666666666666666666666666666666666666666666666666666666666666666666666666666666666666666666666666666666666666666666666666666666666666666666666666666666666666666666666666666666666666666666666666666666666666666666666666666666
               000000000000000000000000000000000000000000000000000000000000000000000000000001111111111111111111111111111111111111111111111111111111111111111111111111111111111111111111111111111111111222222222222222222222222222222222222222222222222222222222222222222222222222222222222222222222222222223333333333333333333333333333333333333333333333333333333333333333333333333333333333333333333333333334
               222222233333333334444444444555555555566666666667777777777888888888899999999990000000000111111111122222222223333333333444444444455555555556666666666777777777788888888889999999999000000000011111111112222222222333333333344444444445555555555666666666677777777778888888888999999999900000000001111111111222222222233333333334444444444555555555566666666667777777777888888888899999999990
               345678901234567890123456789012345678901234567890123456789012345678901234567890123456789012345678901234567890123456789012345678901234567890123456789012345678901234567890123456789012345678901234567890123456789012345678901234567890123456789012345678901234567890123456789012345678901234567890123456789012345678901234567890123456789012345678901234567890123456789012345678901234567890 PCR1
               GTTCTTTCATGGGGAAGCAGATTTGGGTACCACCCAAGTATTGACTCACCCATCAACAACCGCTATGTATTTCGTACATTACTGCCAGCCACCATGAATATTGTACGGTACCATAAATACTTGACCACCTGTAGTACATAAAAACCCAATCCACATCAAAACCCCCTCCCCATGCTTACAAGCAAGTACAGCAATCAACCCTCAACTATCACACATCAACTGCAACTCCAAAGCCACCCCTCACCCACTAGGATACCAACAAACCTACCCACCCTTAACAGTACATAGTACATAAAGCCATTTACCGTACATAGCACATTACAGTCAAATCCCTTCTCGTCCCCATGGATGACCCCCCTCAGATAGGGGTCCCTTGAC
Clone 1 PCR1                              L16055................................................................................................................................T....C............................H16218
Clone 2 PCR1                                    ....................................................................................................................................TCT...........................
Clone 3 PCR1                                    .....................................................................................................................................C............................
Clone 4 PCR1                                    .....................................................................................................................................C............................
Clone 5 PCR1                                    .....................................................................................................................................C............................
Clone 6 PCR1                                    .......................T..................................T......................................................................T...C............................
Clone 7 PCR1                                    .....................................................................................................................................C............................
Clone 8 PCR1                                    ..........T.............................................................................................G............................C.......T....................
Clone 9 PCR1                                    .......................................T...........................................................................................T.C............................
Clone 1 PCR2                                                                                                                                                                                    L16185T..C...............................TTT......................................................T...............................................................................T...................H16378
Clone 2 PCR2                                                                                                                                                                                          .....................................T......................................................T...............................................................................T...................
Clone 3 PCR2                                                                                                                                                                                          ...C.................................T......................................................T...............................................................................T...................
Clone 4 PCR2                                                                                                                                                                                          ...C.................................T......................................................T...............................................................................T...................
Clone 5 PCR2                                                                                                                                                                                          ...C.................................T......................................................T...............................................................................T...................
Clone 6 PCR2                                                                                                                                                                                          ...C.................................T......................................................T...................................................................................................
Clone 7 PCR2                                                                                                                                                                                          ...C.................................T........................................T.............T...............................................................................T...................
Clone 8 PCR2                                                                                                                                                                                          ...C.................................T......................................................T...............................................................................T...................
Clone 9 PCR2                                                                                                                                                                                          ...C.................................T......................................................T...............................................................................T...................
Clone 10 PCR2                                                                                                                                                                                         ...C.................................T......................................................T...........................T...................................................T...................
Clone 11 PCR2                                                                                                                                                                                         ...C.................................T......................................................T...............................................................................T...................

Mirador6 189 223 278 X*

               111111111111111111111111111111111111111111111111111111111111111111111111111111111111111111111111111111111111111111111111111111111111111111111111111111111111111111111111111111111111111111111111111111111111111111111111111111111111111111111111111111111111111111111111111111111111111111111111111111111111111111111111111111111111111111111111111111111111111111111111111111111111111111
               666666666666666666666666666666666666666666666666666666666666666666666666666666666666666666666666666666666666666666666666666666666666666666666666666666666666666666666666666666666666666666666666666666666666666666666666666666666666666666666666666666666666666666666666666666666666666666666666666666666666666666666666666666666666666666666666666666666666666666666666666666666666666666
               000000000000000000000000000000000000000000000000000000000000000000000000000001111111111111111111111111111111111111111111111111111111111111111111111111111111111111111111111111111111111222222222222222222222222222222222222222222222222222222222222222222222222222222222222222222222222222223333333333333333333333333333333333333333333333333333333333333333333333333333333333333333333333333334
               222222233333333334444444444555555555566666666667777777777888888888899999999990000000000111111111122222222223333333333444444444455555555556666666666777777777788888888889999999999000000000011111111112222222222333333333344444444445555555555666666666677777777778888888888999999999900000000001111111111222222222233333333334444444444555555555566666666667777777777888888888899999999990
               345678901234567890123456789012345678901234567890123456789012345678901234567890123456789012345678901234567890123456789012345678901234567890123456789012345678901234567890123456789012345678901234567890123456789012345678901234567890123456789012345678901234567890123456789012345678901234567890123456789012345678901234567890123456789012345678901234567890123456789012345678901234567890 PCR1
               GTTCTTTCATGGGGAAGCAGATTTGGGTACCACCCAAGTATTGACTCACCCATCAACAACCGCTATGTATTTCGTACATTACTGCCAGCCACCATGAATATTGTACGGTACCATAAATACTTGACCACCTGTAGTACATAAAAACCCAATCCACATCAAAACCCCCTCCCCATGCTTACAAGCAAGTACAGCAATCAACCCTCAACTATCACACATCAACTGCAACTCCAAAGCCACCCCTCACCCACTAGGATACCAACAAACCTACCCACCCTTAACAGTACATAGTACATAAAGCCATTTACCGTACATAGCACATTACAGTCAAATCCCTTCTCGTCCCCATGGATGACCCCCCTCAGATAGGGGTCCCTTGAC
Clone 1 PCR1                              L16055..........................T....................................................................................T.....................C............................H16218
Clone 2 PCR1                                    ..........................................................T........................................................................TTT..T.........................
Clone 3 PCR1                                    .......................................N..............................C..............................................................C............................
Clone 4 PCR1                                    ..............................................................................T...................................................T..C............................
Clone 5 PCR1                                    ..............C......................................................................................................................C............................
Clone 6 PCR1                                    ..............C.............................................................................T........................................C............................
Clone 7 PCR1                                    .............T.....................................................................................................................T.C............................
Clone 8 PCR1                                    ..............C......................................................................................................................C............................
Clone 9 PCR1                                    .......................................TT............................................................................................C............................
Clone 10 PCR1                                   .......................................N...................................................................................T....TT.T.C............................
Clone 11 PCR1                                   ..............C......................................................................................................................C............................
Clone 12 PCR1                                   ..............C........................N.............................................................................................C............................
Clone 13 PCR1                                   ..............C....................................................................................................................T.C............................
Clone 1 PCR2                              L16055....................................................................................................A................................C............................H16218
Clone 2 PCR2                                    .....................................................................................................................................C............................
Clone 3 PCR2                                    ...........................................T.........................................................................................C............................
Clone 4 PCR2                                    ..........................................................T..........................................................................C...-........................
Clone 5 PCR2                                    .T.....................................C..........................................................................................TT.C...-........................
Clone 6 PCR2                                    ...........................T...........C.............................................................................................C...-........................
Clone 8 PCR2                                    .......................................T.............................................................................................C....CC......................
Clone 9 PCR2                                    .....................................................................................................................................C............................
Clone 10 PCR2                                   .....................................................................................................................................C............................
Clone 11 PCR2                                   ...........T.................T...........................C..................C...A............C.........................C.............C............................
Clone 1 PCR3                                                                                                                                                                                    L16185...C.................................T......................................................T...................................................................................................H16378
Clone 2 PCR3                                                                                                                                                                                          ...C.................................T......................................................T...................................................................................................
Clone 3 PCR3                                                                                                                                                                                          ...C.................................T......................................................T...................................................................................................
Clone 4 PCR3                                                                                                                                                                                          ...C.................................T......................................................T...................................................................................................

Mirador8 126 294 296 304 T2b*

               111111111111111111111111111111111111111111111111111111111111111111111111111111111111111111111111111111111111111111111111111111111111111111111111111111111111111111111111111111111111111111111111111111111111111111111111111111111111111111111111111111111111111111111111111111111111111111111111111111111111111111111111111111111111111111111111111111111111111111111111111111111111111111
               666666666666666666666666666666666666666666666666666666666666666666666666666666666666666666666666666666666666666666666666666666666666666666666666666666666666666666666666666666666666666666666666666666666666666666666666666666666666666666666666666666666666666666666666666666666666666666666666666666666666666666666666666666666666666666666666666666666666666666666666666666666666666666
               000000000000000000000000000000000000000000000000000000000000000000000000000001111111111111111111111111111111111111111111111111111111111111111111111111111111111111111111111111111111111222222222222222222222222222222222222222222222222222222222222222222222222222222222222222222222222222223333333333333333333333333333333333333333333333333333333333333333333333333333333333333333333333333334
               222222233333333334444444444555555555566666666667777777777888888888899999999990000000000111111111122222222223333333333444444444455555555556666666666777777777788888888889999999999000000000011111111112222222222333333333344444444445555555555666666666677777777778888888888999999999900000000001111111111222222222233333333334444444444555555555566666666667777777777888888888899999999990
               345678901234567890123456789012345678901234567890123456789012345678901234567890123456789012345678901234567890123456789012345678901234567890123456789012345678901234567890123456789012345678901234567890123456789012345678901234567890123456789012345678901234567890123456789012345678901234567890123456789012345678901234567890123456789012345678901234567890123456789012345678901234567890 PCR1
               GTTCTTTCATGGGGAAGCAGATTTGGGTACCACCCAAGTATTGACTCACCCATCAACAACCGCTATGTATTTCGTACATTACTGCCAGCCACCATGAATATTGTACGGTACCATAAATACTTGACCACCTGTAGTACATAAAAACCCAATCCACATCAAAACCCCCTCCCCATGCTTACAAGCAAGTACAGCAATCAACCCTCAACTATCACACATCAACTGCAACTCCAAAGCCACCCCTCACCCACTAGGATACCAACAAACCTACCCACCCTTAACAGTACATAGTACATAAAGCCATTTACCGTACATAGCACATTACAGTCAAATCCCTTCTCGTCCCCATGGATGACCCCCCTCAGATAGGGGTCCCTTGAC
Clone 1(L16995).......................................................C...............................................C.....H16132
Clone 2 PCR1   .......................................................................................................C.....
Clone 3 PCR1   .......................................................................................................C.....
Clone 4 PCR1   .........................................................................A...........TC................C.....
Clone 5 PCR1   ..............................T.T......................................................................C.....
Clone 6 PCR1   ...............................G.......................................................................C.....
Clone 1 PCR2                              L16055......................................................................C...........................................................................................H16218
Clone 2 PCR2                                    ......................................................................C...........................................................................................
Clone 3 PCR2                                    ......................................................................C.........................................................T.................................
Clone 4 PCR2                                    .............T..TT....................................................C...........................................................................................
Clone 5 PCR2                                    ......................................................................C...........................................................................................
Clone 6 PCR2                                    ......................................................................C.................................................T.........................................
Clone 7 PCR2                                    ......................................................................C...........................................................................................
Clone 7 PCR2                                    ......................................................................C...........................................................................................
Clone 9 PCR2                                    ...............T......................................................C.T.........................................................................................
Clone 10 PCR2                                   ......................................................................C...........................................................................................
Clone 11 PCR2                                   ......................................................................C...........................................................................................
Clone 12 PCR2                                   ......................................................................C...........................................................................................
Clone 13 PCR2                                   ......................................................................C...........................................................................................
Clone 1 PCR3                                                                                              L16107..................C........................................................................................................................H16247
Clone 2 PCR3                                                                                                    ..................C........................................................................................................................
Clone 3 PCR3                                                                                                    ..................C......................N.................................................................................................
Clone 4 PCR3                                                                                                    ..................C........................................................................................................................
Clone 5 PCR3                                                                                                    ..................C........................................TT....T.........................................................................
Clone 6 PCR3                                                                                                    ..................C........................................................................................................................
Clone 7 PCR3                                                                                                    ..................C........................................................................................................................
Clone 8 PCR3                                                                                                    ..................C........................................................................................................................
Clone 9 PCR3                                                                                                    ..................C........................................................................................................................
Clone 1 PCR4                                                                                                                                                                                    L16185........................................................................................................T...T.T.......C.........................................................................H16378
Clone 2 PCR4                                                                                                                                                                                          ............................................................................................................T.T.......C.........................................................................
Clone 3 PCR4                                                                                                                                                                                          ............................................................................................................T.T.......C.........................................................................
Clone 4 PCR4                                                                                                                                                                                          ............................................................................................................T.T.......C.........................................................................
Clone 5 PCR4                                                                                                                                                                                          ............................................................................................................T.T.......C.........................................................................
Clone 6 PCR4                                                                                                                                                                                          ............................................................................................................T.T.......C.........................................................................
Clone 7 PCR4                                                                                                                                                                                          ............................................................................................................T.T.......C...............................................C.........................
Clone 8 PCR4                                                                                                                                                                                          ............................................................................................................T.T.......C.........................................................................
Clone 9 PCR4                                                                                                                                                                                          ............................................................................................................T.T.......C..................................................T......................
Clone 10 PCR4                                                                                                                                                                                         ............................................................................................................T.T.......C.........................................................................
Clone 11 PCR4                                                                                                                                                                                         ............................................................................................................T.T.......C.........................................................................
Clone 12 PCR4                                                                                                                                                                                         ............................................................................................................T.T.......C.........................................................................
Clone 13 PCR4                                                                                                                                                                                         ............................................................................................................T.T.......C.........................................................................
Clone 14 PCR4                                                                                                                                                                                         ............................................................................................................T.T....T..C.........................................................................
Clone 15 PCR4                                                                                                                                                                                         ............................................................................................................T.T.......C......C..................................................................
Clone 16 PCR4                                                                                                                                                                                         ....................................................................................................TT......T.T.......C..............A..........................................................
Clone 1 PCR5                                                                                                                                                                                                                         L16222.......................................................................T.T.......C......................H16327
Clone 2 PCR5                                                                                                                                                                                                                               ...........................TT....T.....................................T.T.......C......................
Clone 3 PCR5                                                                                                                                                                                                                               .......................................................................T.T.......C......................
Clone 4 PCR5                                                                                                                                                                                                                               .......................................................................T.T.......C......................
Clone 5 PCR5                                                                                                                                                                                                                               .......................................................................T.T.......C......................
Clone 6 PCR5                                                                                                                                                                                                                               .......................................................................T.T.......C......................
Clone 7 PCR5                                                                                                                                                                                                                               ...............................................T.......................T.T.......C......................
```

```
Clone 8 PCR5                                                                                                                                                                                                         ...........................TT....T.....................................T.T.......C......................

Mirador9 189 223 278 K*

              111111111111111111111111111111111111111111111111111111111111111111111111111111111111111111111111111111111111111111111111111111111111111111111111111111111111111111111111111111111111111111111111111111111111111111111111111111111111111111111111111111111111111111111111111111111111111111111111111111111111111111111111111111111111111111111111111111111111111111111111111111111111
              666666666666666666666666666666666666666666666666666666666666666666666666666666666666666666666666666666666666666666666666666666666666666666666666666666666666666666666666666666666666666666666666666666666666666666666666666666666666666666666666666666666666666666666666666666666666666666666666666666666666666666666666666666666666666666666666666666666666666666666666666666666666
              000000000000000000000000000000000000000000000000000000000000000000000000000001111111111111111111111111111111111111111111111111111111111111111111111111111111111111111111111111111111122222222222222222222222222222222222222222222222222222222222222222222222222222222222222222222222222222222223333333333333333333333333333333333333333333333333333333333333333333333333333333333333333333333333334
              222222233333333334444444444555555555566666666667777777777888888888899999999990000000000111111111122222222223333333333444444444455555555556666666666777777777788888888889999999999000000000011111111112222222222333333333344444444445555555555666666666677777777778888888888999999999900000000001111111111222222222233333333334444444444555555555566666666667777777777888888888899999999990
              345678901234567890123456789012345678901234567890123456789012345678901234567890123456789012345678901234567890123456789012345678901234567890123456789012345678901234567890123456789012345678901234567890123456789012345678901234567890123456789012345678901234567890123456789012345678901234567890123456789012345678901234567890123456789012345678901234567890123456789012345678901234567890 PCR1
              GTTCTTTCATGGGGAAGCAGATTTGGGTACCACCCAAGTATTGACTCACCCATCAACAACCGCTATGTATTTCGTACATTACTGCCAGCCACCATGAATATTGTACGGTACCATAAATACTTGACCACCTGTAGTACATAAAAACCCAATCCACATCAAAACCCCCTCCCCATGCTTACAAGCAAGTACAGCAATCAACCCTCAACTATCACACATCAACTGCAACTCCAAAGCCACCCCTCACCCACTAGGATACCAACAAACCTACCCACCCTTAACAGTACATAGTACATAAAGCCATTTACCGTACATAGCACATTACAGTCAAATCCCTTCTCGTCCCCATGGATGACCCCCCTCAGATAGGGGTCCCTTGAC
Clone 1 PCR1                             L16055.................................................................................................................................TT...............................H16218 PCR1
Clone 2 PCR1                                   ............................................................................................T.....................................T..C............................
Clone 3 PCR1                                   ..................................................................................................................................T..C............................
Clone 4 PCR1                                   ...............T........................................................T............................................TT..............CT............GA.............
Clone 5 PCR1                                   .................................................................................................................................TTT.C............................
Clone 6 PCR1                                   ...........A.................G..................A......G.....C.......................................................T............T.TCT.T....T....................
Clone 7 PCR1                                   ..............................................................A......A...A...........................................................C.G..........................
Clone 8 PCR1                                   .....................................................................................................................................C............G..G............
Clone 9 PCR1                                   ....................T......................T.....................................................................................T...C............................
Clone 10 PCR1                                  ................................................................................................................................AA.T.CTT.T........................
Clone 11 PCR1                                  .....................................................................................................................................C............................
Clone 12 PCR1                                  ..........................................................T..........................................................................C............................
Clone 1 PCR2                                                                                                                                                                                   L16185...C...-...T.........................T....T.................................................T...................................................................................GT...........TT.H16378
Clone 3 PCR2                                                                                                                                                                                         ..TC.................................T..................................T...................T...................................................................................................
Clone 4 PCR2                                                                                                                                                                                         ...C.................................T......................................................T...................................................................................................
Clone 5 PCR2                                                                                                                                                                                         ...C.................................T.........................................T............T................................................................................................TT.
Clone 6 PCR2                                                                                                                                                                                         ...C.................................T......................................................T..........................T........................................................................
Clone 7 PCR2                                                                                                                                                                                         ...C.................................T......................................................T..........................T..............................T...........................T.............
Clone 8 PCR2                                                                                                                                                                                         ...C.................................T......................................................T................................................................T.................................T
Clone 9 PCR2                                                                                                                                                                                         ...C.................................T......................................................T..........................T.....................................................................T.T
Clone 10 PCR2                                                                                                                                                                                        ...C.......G.........................T....A.............G...................................T..........................T.......................................................................T

Mirador10 224 256 311 K*

              111111111111111111111111111111111111111111111111111111111111111111111111111111111111111111111111111111111111111111111111111111111111111111111111111111111111111111111111111111111111111111111111111111111111111111111111111111111111111111111111111111111111111111111111111111111111111111111111111111111111111111111111111111111111111111111111111111111111111111111111111111111111
              666666666666666666666666666666666666666666666666666666666666666666666666666666666666666666666666666666666666666666666666666666666666666666666666666666666666666666666666666666666666666666666666666666666666666666666666666666666666666666666666666666666666666666666666666666666666666666666666666666666666666666666666666666666666666666666666666666666666666666666666666666666666
              000000000000000000000000000000000000000000000000000000000000000000000000000001111111111111111111111111111111111111111111111111111111111111111111111111111111111111111111111111111111122222222222222222222222222222222222222222222222222222222222222222222222222222222222222222222222222222222223333333333333333333333333333333333333333333333333333333333333333333333333333333333333333333333333334
              222222233333333334444444444555555555566666666667777777777888888888899999999990000000000111111111122222222223333333333444444444455555555556666666666777777777788888888889999999999000000000011111111112222222222333333333344444444445555555555666666666677777777778888888888999999999900000000001111111111222222222233333333334444444444555555555566666666667777777777888888888899999999990
              345678901234567890123456789012345678901234567890123456789012345678901234567890123456789012345678901234567890123456789012345678901234567890123456789012345678901234567890123456789012345678901234567890123456789012345678901234567890123456789012345678901234567890123456789012345678901234567890123456789012345678901234567890123456789012345678901234567890123456789012345678901234567890 PCR1
              GTTCTTTCATGGGGAAGCAGATTTGGGTACCACCCAAGTATTGACTCACCCATCAACAACCGCTATGTATTTCGTACATTACTGCCAGCCACCATGAATATTGTACGGTACCATAAATACTTGACCACCTGTAGTACATAAAAACCCAATCCACATCAAAACCCCCTCCCCATGCTTACAAGCAAGTACAGCAATCAACCCTCAACTATCACACATCAACTGCAACTCCAAAGCCACCCCTCACCCACTAGGATACCAACAAACCTACCCACCCTTAACAGTACATAGTACATAAAGCCATTTACCGTACATAGCACATTACAGTCAAATCCCTTCTCGTCCCCATGGATGACCCCCCTCAGATAGGGGTCCCTTGAC
Clone 1 PCR1                             L16055......................................................................................T............................................T..............................H16218 PCR1
Clone 2 PCR1                                   ..............C......................C..............................................C.............................................................................
Clone 3 PCR1                                   ..................................................................................................................................................................
Clone 4 PCR1                                   ..................................................................................................................................................................
Clone 5 PCR1                                   ..................................................................................................................................................................
Clone 6 PCR1                                   ..................................................................................................................................................................
Clone 7 PCR1                                   ...............................................................................................................................-..................................
Clone 8 PCR1                                   ..................................................................................................................................................................
Clone 9 PCR1                                   ..........................T.......................................................................................................................................
Clone 10 PCR1                                  ..................................................................................................................................................................
Clone 11 PCR1                                  .............................G....................................................................................................................................
Clone 1 PCR2                                                                                                                                                                                   L16185......................................C...T...........................T......................................................C.................................................................TH16378
Clone 2 PCR2                                                                                                                                                                                         T.....................................C...............................T......................................................C.................................................................T
Clone 3 PCR2                                                                                                                                                                                         ...........T..........................C..........................TT...T......................................................C..................................................................
Clone 4 PCR2                                                                                                                                                                                         ......................................C...............................T..........................T...........................C...............................................................T..
Clone 5 PCR2                                                                                                                                                                                         ......................................C...............................T......................................................C..................................................................
Clone 6 PCR2                                                                                                                                                                                         ......................................C...............................T......................................................C................................................................TT
Clone 7 PCR2                                                                                                                                                                                         ......................................C...............................T.......T.....T.......................T................C...............................................................T..
Clone 8 PCR2                                                                                                                                                                                         ......................................C...............................T......................................................C...............................................................T..
Clone 9 PCR2                                                                                                                                                                                         ......................................C...............................T.......T..............................................C................................................................TT
Clone 10 PCR2                                                                                                                                                                                        ......................................C...........T...................T......................................................C................................................................T.
Clone 11 PCR2                                                                                                                                                                                        ...........G..........................C...............................T......................................................C..................................................................
Clone 12 PCR2                                                                                                                                                                                        ......................................C...............................T......................................................C..................................................................
Clone 13 PCR2                                                                                                                                                                                        ......................................C...............................T......................................................C..............................................T...................

Mirador11 CRS

              111111111111111111111111111111111111111111111111111111111111111111111111111111111111111111111111111111111111111111111111111111111111111111111111111111111111111111111111111111111111111111111111111111111111111111111111111111111111111111111111111111111111111111111111111111111111111111111111111111111111111111111111111111111111111111111111111111111111111111111111111111111111
              666666666666666666666666666666666666666666666666666666666666666666666666666666666666666666666666666666666666666666666666666666666666666666666666666666666666666666666666666666666666666666666666666666666666666666666666666666666666666666666666666666666666666666666666666666666666666666666666666666666666666666666666666666666666666666666666666666666666666666666666666666666666
              000000000000000000000000000000000000000000000000000000000000000000000000000001111111111111111111111111111111111111111111111111111111111111111111111111111111111111111111111111111111122222222222222222222222222222222222222222222222222222222222222222222222222222222222222222222222222222222223333333333333333333333333333333333333333333333333333333333333333333333333333333333333333333333333334
              222222233333333334444444444555555555566666666667777777777888888888899999999990000000000111111111122222222223333333333444444444455555555556666666666777777777788888888889999999999000000000011111111112222222222333333333344444444445555555555666666666677777777778888888888999999999900000000001111111111222222222233333333334444444444555555555566666666667777777777888888888899999999990
              345678901234567890123456789012345678901234567890123456789012345678901234567890123456789012345678901234567890123456789012345678901234567890123456789012345678901234567890123456789012345678901234567890123456789012345678901234567890123456789012345678901234567890123456789012345678901234567890123456789012345678901234567890123456789012345678901234567890123456789012345678901234567890 PCR1
              GTTCTTTCATGGGGAAGCAGATTTGGGTACCACCCAAGTATTGACTCACCCATCAACAACCGCTATGTATTTCGTACATTACTGCCAGCCACCATGAATATTGTACGGTACCATAAATACTTGACCACCTGTAGTACATAAAAACCCAATCCACATCAAAACCCCCTCCCCATGCTTACAAGCAAGTACAGCAATCAACCCTCAACTATCACACATCAACTGCAACTCCAAAGCCACCCCTCACCCACTAGGATACCAACAAACCTACCCACCCTTAACAGTACATAGTACATAAAGCCATTTACCGTACATAGCACATTACAGTCAAATCCCTTCTCGTCCCCATGGATGACCCCCCTCAGATAGGGGTCCCTTGAC
Clone 1 PCR1                             L16055..................................................................................................................................T..T............................H16218 PCR1
Clone 2 PCR1                                   ..................................................................................................................................................................
Clone 3 PCR1                                   ..................................................................................................................................................................
Clone 4 PCR1                                   .............................G....................................................................................................................................
Clone 5 PCR1                                   ...........................................................................................TT.....................................................................
Clone 6 PCR1                                   ................................................................................................C.................................................................
Clone 7 PCR1                                   ..................................................................................................................................................................
Clone 8 PCR1                                   ............................................................................................T.TT..................................................................
Clone 9 PCR1                                   ..................................................................................................................................................................
Clone 10 PCR1                                  .......................T....................................................................T.....................................................................
Clone 11 PCR1                                  ...................................................................................................................................................G..............
Clone 12 PCR1                                  ..................................................................................................................................................................
Clone 13 PCR1                                  ..................................................................................................................................................T...............
Clone 14 PCR1                                  ..................................................................................................................................................................
Clone 15 PCR1                                  ..................................................................................................................................................................
Clone 16 PCR1                                  ...............TT...............................T..................................................................................T..............................
Clone 17 PCR1                                  TT..................................................T...........................................................................T....C............................
Clone 18 PCR1                                  .............T....................................................................................................................................................
Clone 19 PCR1                                  ................................................................................................................................................TT................
Clone 20 PCR1                                  ..................................................................................................................................................................
Clone 21 PCR1                                  ...........A......................................................................................................................................................
Clone 22 PCR1                                  ..............................................................................T...............T...................................................................
Clone 1 PCR2                                                                                                                                                                                   L16185......................................................................................................................................................................................G.........H16378
Clone 2 PCR2                                                                                                                                                                                         ................................................................................................................................................................................................
Clone 3 PCR2                                                                                                                                                                                         ................................................................................................................................................................................................
Clone 4 PCR2                                                                                                                                                                                         ................................................................................................................................................................................................
Clone 5 PCR2                                                                                                                                                                                         ................................................................................................................................................................................................
Clone 6 PCR2                                                                                                                                                                                         .............................................................................................................................................................................................T..
Clone 7 PCR2                                                                                                                                                                                         ...............................................................................................................................T..............................................................T.
Clone 8 PCR2                                                                                                                                                                                         ..............................................................................T.......................................................................................................G.......T.
Clone 9 PCR2                                                                                                                                                                                         ................................................................................................................................................................................................
Clone 10 PCR2                                                                                                                                                                                        ............................................................................................................T...................................................................................
Clone 11 PCR2                                                                                                                                                                                        ................................................................................................................................................................................................
Clone 12 PCR2                                                                                                                                                                                        ............................................................G...................................................................................................................................
Clone 13 PCR2                                                                                                                                                                                        ................................................................................................................................................................................................
Clone 14 PCR2                                                                                                                                                                                        ......................................................................................................................................................................................G.A...A...
Clone 15 PCR2                                                                                                                                                                                        ................................................................................................................................................................................................
Clone 16 PCR2                                                                                                                                                                                        .......................................................................................................................................................................T.......T................
Clone 17 PCR2                                                                                                                                                                                        ...............................................................................................................................................................................................T
Clone 18 PCR2                                                                                                                                                                                        ....................................................................................A.................................................................................................G.........

Mirador12 224 311 K*

              111111111111111111111111111111111111111111111111111111111111111111111111111111111111111111111111111111111111111111111111111111111111111111111111111111111111111111111111111111111111111111111111111111111111111111111111111111111111111111111111111111111111111111111111111111111111111111111111111111111111111111111111111111111111111111111111111111111111111111111111111111111111
              666666666666666666666666666666666666666666666666666666666666666666666666666666666666666666666666666666666666666666666666666666666666666666666666666666666666666666666666666666666666666666666666666666666666666666666666666666666666666666666666666666666666666666666666666666666666666666666666666666666666666666666666666666666666666666666666666666666666666666666666666666666666
              000000000000000000000000000000000000000000000000000000000000000000000000000001111111111111111111111111111111111111111111111111111111111111111111111111111111111111111111111111111111122222222222222222222222222222222222222222222222222222222222222222222222222222222222222222222222222222222223333333333333333333333333333333333333333333333333333333333333333333333333333333333333333333333333334
              222222233333333334444444444555555555566666666667777777777888888888899999999990000000000111111111122222222223333333333444444444455555555556666666666777777777788888888889999999999000000000011111111112222222222333333333344444444445555555555666666666677777777778888888888999999999900000000001111111111222222222233333333334444444444555555555566666666667777777777888888888899999999990
              345678901234567890123456789012345678901234567890123456789012345678901234567890123456789012345678901234567890123456789012345678901234567890123456789012345678901234567890123456789012345678901234567890123456789012345678901234567890123456789012345678901234567890123456789012345678901234567890123456789012345678901234567890123456789012345678901234567890123456789012345678901234567890 PCR1
              GTTCTTTCATGGGGAAGCAGATTTGGGTACCACCCAAGTATTGACTCACCCATCAACAACCGCTATGTATTTCGTACATTACTGCCAGCCACCATGAATATTGTACGGTACCATAAATACTTGACCACCTGTAGTACATAAAAACCCAATCCACATCAAAACCCCCTCCCCATGCTTACAAGCAAGTACAGCAATCAACCCTCAACTATCACACATCAACTGCAACTCCAAAGCCACCCCTCACCCACTAGGATACCAACAAACCTACCCACCCTTAACAGTACATAGTACATAAAGCCATTTACCGTACATAGCACATTACAGTCAAATCCCTTCTCGTCCCCATGGATGACCCCCCTCAGATAGGGGTCCCTTGAC
Clone 1 PCR1                             L16055...................................................................C..............................................................................................
```

```
Clone 2 PCR1                                    ................................................................................................................TT................................................
Clone 3 PCR1                                    ...........................T......................................................................................................................................
Clone 4 PCR1                                    .................................................................................................A..........................................A.....G..........A....
Clone 5 PCR1                                    ..........................................................................................G.......................................................G...............
Clone 6 PCR1                                    ........................................................................................................................T.........................................
Clone 7 PCR1                                    ..................................................................................................................................................................
Clone 8 PCR1                                    ..................................................................................................................................................................
Clone 9 PCR1                                    .........................................................................A.............................T..........................................................
Clone 10 PCR1                                   ........................................A...........................................................A...................................T.........................
Clone 11 PCR1                                   ........................................................................................................................T..T......................................
Clone 12 PCR1                                   ............................................................................................................................................A.....................
Clone 13 PCR1                                   ..................................................................................................................................................................
Clone 14 PCR1                                   ..................................................................................................................................................................
Clone 15 PCR1                                   .................................................................................................................T...T............................................
Clone 16 PCR1                                   .............T....................................................................................................................................................
Clone 1 PCR2                                                                                                                                                                          L16185.......T.....................................................................................T...............................C..................................................................H16378
Clone 2 PCR2                                                                                                                                                                                ......................................C......................................................................................C........................................................C.........
Clone 3 PCR2                                                                                                                                                                                .......................C..............C.........................................T............................................C..................................................................
Clone 4 PCR2                                                                                                                                                                                TT....................................C......................................................................................C..................................................................
Clone 5 PCR2                                                                                                                                                                                ......................................C......................................................................................C..................................................................
Clone 6 PCR2                                                                                                                                                                                TT....................................C.................A....................................................................C..................................................................
Clone 7 PCR2                                                                                                                                                                                ......................................C................................T..TT........T........................................C..................................................................
Clone 8 PCR2                                                                                                                                                                                .......................C..............C.........................................T............................................C..................................................................
Clone 9 PCR2                                                                                                                                                                                ......................................C.......................................................G..............................C..................................................................
Clone 10 PCR2                                                                                                                                                                               .................G...................T..................................................................T.......................................................................................
Clone 11 PCR2                                                                                                                                                                               ......................................C......................................................................................C................................T...............T...T............T
Clone 12 PCR2                                                                                                                                                                               ......................................C......................................................................................C..................................................................
Clone 13 PCR2                                                                                                                                                                               ......................................C.............................................T.................................C.........................................................................
Clone 14 PCR2                                                                                                                                                                               .....................................T..................................................................T.......................................................................................
Clone 15 PCR2                                                                                                                                                                               .............................................................................................................................C..................................................................
Clone 16 PCR2                                                                                                                                                                               ......T.............................................................................T.................................C...............................................................N.........

Mirador13 278 H*

               111111111111111111111111111111111111111111111111111111111111111111111111111111111111111111111111111111111111111111111111111111111111111111111111111111111111111111111111111111111111111111111111111111111111111111111111111111111111111111111111111111111111111111111111111111111111111111111111111111111111111111111111111111111111111111111111111111111111111111111111111111111111
               666666666666666666666666666666666666666666666666666666666666666666666666666666666666666666666666666666666666666666666666666666666666666666666666666666666666666666666666666666666666666666666666666666666666666666666666666666666666666666666666666666666666666666666666666666666666666666666666666666666666666666666666666666666666666666666666666666666666666666666666666666666666
               000000000000000000000000000000000000000000000000000000000000000000000000000001111111111111111111111111111111111111111111111111111111111111111111111111111111111111111111111111111111111111111111111111111111112222222222222222222222222222222222222222222222222222222222222222222222222222222222222222222222222222222222222222222222222222222222222222222222222222222222222222222222223333333333333333333333333333333333333333333333333333333333333333333333333333333333333333333333333333333333333334
               222222233333333334444444444555555555566666666667777777777888888888899999999990000000000111111111122222222223333333333444444444455555555556666666666777777777788888888889999999999000000000011111111112222222222333333333344444444445555555555666666666677777777778888888888999999999900000000001111111111222222222233333333334444444444555555555566666666667777777777888888888899999999990
               345678901234567890123456789012345678901234567890123456789012345678901234567890123456789012345678901234567890123456789012345678901234567890123456789012345678901234567890123456789012345678901234567890123456789012345678901234567890123456789012345678901234567890123456789012345678901234567890123456789012345678901234567890123456789012345678901234567890123456789012345678901234567890 PCR1
               GTTCTTTCATGGGGAAGCAGATTTGGGTACCACCCAAGTATTGACTCACCCATCAACAACCGCTATGTATTTCGTACATTACTGCCAGCCACCATGAATATTGTACGGTACCATAAATACTTGACCACCTGTAGTACATAAAAACCCAATCCACATCAAAACCCCCTCCCCATGCTTACAAGCAAGTACAGCAATCAACCCTCAACTATCACACATCAACTGCAACTCCAAAGCCACCCCTCACCCACTAGGATACCAACAAACCTACCCACCCTTAACAGTACATAGTACATAAAGCCATTTACCGTACATAGCACATTACAGTCAAATCCCTTCTCGTCCCCATGGATGACCCCCCTCAGATAGGGGTCCCTTGAC
Clone 1 PCR1                              L16055.....................................C....................................................................................................................G.......H16218
Clone 2 PCR1                                    ..................................................................................................................................................................
Clone 3 PCR1                                    ..................................................A...................................T...........................................................................
Clone 4 PCR1                                    ..................................................................................................................................................................
Clone 5 PCR1                                    ......................................................................C...........................................................................................
Clone 6 PCR1                                    ...........................................T............T.TT......................................................................................................
Clone 7 PCR1                                    ...................................................................................................................................T..............................
Clone 8 PCR1                                    ..............................................................................................................................................A...................
Clone 9 PCR1                                    ............................................................................................T..................T..................................................
Clone 10 PCR1                                   ..................................................................................................................................................................
Clone 11 PCR1                                   ...........................................................................................................................................................T......
Clone 12 PCR1                                   ..................................................................................................................................................................
Clone 13 PCR1                                   .................................A................................................................................................................................
Clone 14 PCR1                                   ..................................................................................................................................................G...............
Clone 15 PCR1                                   ..................................................................................................................................................................
Clone 16 PCR1                                   .....................................C............................................................................................................................
Clone 1 PCR2                                                                                                                                                                          L16185............................................................................................T...................................................................................................H16378
Clone 2 PCR2                                                                                                                                                                                .........C..................................................................................T...................................................................................................
Clone 3 PCR2                                                                                                                                                                                .............................................C..............................................T...................................................................................................
Clone 4 PCR2                                                                                                                                                                                ............................................................................................T............T......................................................................................
Clone 5 PCR2                                                                                                                                                                                ............................................................................................T.................................................T........................T....T...................
Clone 6 PCR2                                                                                                                                                                                ............................................................................................T...................................................................................................
Clone 7 PCR2                                                                                                                                                                                ........................................................................................A...T...................................................................................................
Clone 8 PCR2                                                                                                                                                                                ............................................................................................T...................................................................................................
Clone 9 PCR2                                                                                                                                                                                ...........................A................................................................T..................................T...........................................................A....
Clone 10 PCR2                                                                                                                                                                               ............................................................................................T...................................................................................................
Clone 11 PCR2                                                                                                                                                                               ............................................................................T.T.............T...................................................................................................
Clone 12 PCR2                                                                                                                                                                               ............................................................................................T...................................................................................................
Clone 13 PCR2                                                                                                                                                                               .......T............................T...................T...................................T................................................T..................................................
Clone 14 PCR2                                                                                                                                                                               .T.....T....................................................................................T...................................................................................................
Clone 15 PCR2                                                                                                                                                                               ............................................................................................T...................................................................................................
Clone 16 PCR2                                                                                                                                                                               ...............NNN..........................................................................T..............................G....................................................................
Clone 17 PCR2                                                                                                                                                                               .......T........................................................................................................................................................................................

Mirador15 126 294 296 304 T2b*

               111111111111111111111111111111111111111111111111111111111111111111111111111111111111111111111111111111111111111111111111111111111111111111111111111111111111111111111111111111111111111111111111111111111111111111111111111111111111111111111111111111111111111111111111111111111111111111111111111111111111111111111111111111111111111111111111111111111111111111111111111111111111
               666666666666666666666666666666666666666666666666666666666666666666666666666666666666666666666666666666666666666666666666666666666666666666666666666666666666666666666666666666666666666666666666666666666666666666666666666666666666666666666666666666666666666666666666666666666666666666666666666666666666666666666666666666666666666666666666666666666666666666666666666666666666
               000000000000000000000000000000000000000000000000000000000000000000000000000001111111111111111111111111111111111111111111111111111111111111111111111111111111111111111111111111111111111111111111111111111111112222222222222222222222222222222222222222222222222222222222222222222222222222222222222222222222222222222222222222222222222222222222222222222222222222222222222222222222223333333333333333333333333333333333333333333333333333333333333333333333333333333333333333333333333333333333333334
               222222233333333334444444444555555555566666666667777777777888888888899999999990000000000111111111122222222223333333333444444444455555555556666666666777777777788888888889999999999000000000011111111112222222222333333333344444444445555555555666666666677777777778888888888999999999900000000001111111111222222222233333333334444444444555555555566666666667777777777888888888899999999990
               345678901234567890123456789012345678901234567890123456789012345678901234567890123456789012345678901234567890123456789012345678901234567890123456789012345678901234567890123456789012345678901234567890123456789012345678901234567890123456789012345678901234567890123456789012345678901234567890123456789012345678901234567890123456789012345678901234567890123456789012345678901234567890 PCR1
               GTTCTTTCATGGGGAAGCAGATTTGGGTACCACCCAAGTATTGACTCACCCATCAACAACCGCTATGTATTTCGTACATTACTGCCAGCCACCATGAATATTGTACGGTACCATAAATACTTGACCACCTGTAGTACATAAAAACCCAATCCACATCAAAACCCCCTCCCCATGCTTACAAGCAAGTACAGCAATCAACCCTCAACTATCACACATCAACTGCAACTCCAAAGCCACCCCTCACCCACTAGGATACCAACAAACCTACCCACCCTTAACAGTACATAGTACATAAAGCCATTTACCGTACATAGCACATTACAGTCAAATCCCTTCTCGTCCCCATGGATGACCCCCCTCAGATAGGGGTCCCTTGAC
Clone 1 PCR1                              L16055.............T....................................................................................................................................................H16218
Clone 2 PCR1                                    .....................................C.........................................................................................................NNNNN..............
Clone 3 PCR1                                    ......................................................................C...........................................................................................
Clone 4 PCR1                                    ......................................................................C.........................................................................NNN...............
Clone 5 PCR1                                    .....................................C...........................................................................................................NN...............
Clone 6 PCR1                                    ......................................................................C...........................................................................................
Clone 7 PCR1                                    .............T........................................................C..................A...............................................T.....NNNNNN.............
Clone 8 PCR1                                    ......................................................................C...........................................................................T...............
Clone 9 PCR1                                    ......................................................................C........................................T................................NN................
Clone 1 PCR2                              L16055.............T..........................................................................................................................T......................... H16218
Clone 2 PCR2                                    ......................................................................C.......................................................................................T...
Clone 3 PCR2                                    .....................T............................................................................................................................................
Clone 4 PCR2                                    .....................................C............................................................................................................................
Clone 5 PCR2                                    ......................................................................C..................................................................T........................
Clone 6 PCR2                                    .............T........................................................C..................A........................................................................
Clone 7 PCR2                                    ........................................................................................................................................T.........................
Clone 8 PCR2                                    ......................................................................C....................................G.....................TT..C............................
Clone 9 PCR2                                    .......................T..............................................C....................................G......................T..C............................
Clone 10 PCR2                                   .........................................................................A........................................................................................
Clone 1 PCR3                                                                                                                                                                          L16185............................................................................................................T.T.......C.........................................................................H16378
Clone 2 PCR3                                                                                                                                                                                ......................................C.....................................................................T.T.......C.........................................................................
Clone 3 PCR3                                                                                                                                                                                ....................................T......................................T................................T...................................................................................
Clone 4 PCR3                                                                                                                                                                                ............................................................................................................T.T.......C...............................................................C.........
Clone 5 PCR3                                                                                                                                                                                ......T............................T................................................T.................................C.........................................................................
Clone 6 PCR3                                                                                                                                                                                ............................................................................................................T.T.......C.........................................................................
Clone 7 PCR3                                                                                                                                                                                ............................................................................................................T.T.......C.........................................................................
Clone 8 PCR3                                                                                                                                                                                ............................................................................................................T.T.......C.........................................................................
Clone 9 PCR3                                                                                                                                                                                ............................................................................................................T.T.......C.........................................................................
Clone 10 PCR3                                                                                                                                                                               ............................................................................................................T.T.......C..............................G..........................................
Clone 11 PCR3                                                                                                                                                                               ......................................................................................................................C..............................G..........................................

Mirador16 093 224 311 K*

               111111111111111111111111111111111111111111111111111111111111111111111111111111111111111111111111111111111111111111111111111111111111111111111111111111111111111111111111111111111111111111111111111111111111111111111111111111111111111111111111111111111111111111111111111111111111111111111111111111111111111111111111111111111111111111111111111111111111111111111111111111111111
               666666666666666666666666666666666666666666666666666666666666666666666666666666666666666666666666666666666666666666666666666666666666666666666666666666666666666666666666666666666666666666666666666666666666666666666666666666666666666666666666666666666666666666666666666666666666666666666666666666666666666666666666666666666666666666666666666666666666666666666666666666666666
               000000000000000000000000000000000000000000000000000000000000000000000000000001111111111111111111111111111111111111111111111111111111111111111111111111111111111111111111111111111111111111111111111111111111112222222222222222222222222222222222222222222222222222222222222222222222222222222222222222222222222222222222222222222222222222222222222222222222222222222222222222222222223333333333333333333333333333333333333333333333333333333333333333333333333333333333333333333333333333333333333334
               222222233333333334444444444555555555566666666667777777777888888888899999999990000000000111111111122222222223333333333444444444455555555556666666666777777777788888888889999999999000000000011111111112222222222333333333344444444445555555555666666666677777777778888888888999999999900000000001111111111222222222233333333334444444444555555555566666666667777777777888888888899999999990
               345678901234567890123456789012345678901234567890123456789012345678901234567890123456789012345678901234567890123456789012345678901234567890123456789012345678901234567890123456789012345678901234567890123456789012345678901234567890123456789012345678901234567890123456789012345678901234567890123456789012345678901234567890123456789012345678901234567890123456789012345678901234567890 PCR1
               GTTCTTTCATGGGGAAGCAGATTTGGGTACCACCCAAGTATTGACTCACCCATCAACAACCGCTATGTATTTCGTACATTACTGCCAGCCACCATGAATATTGTACGGTACCATAAATACTTGACCACCTGTAGTACATAAAAACCCAATCCACATCAAAACCCCCTCCCCATGCTTACAAGCAAGTACAGCAATCAACCCTCAACTATCACACATCAACTGCAACTCCAAAGCCACCCCTCACCCACTAGGATACCAACAAACCTACCCACCCTTAACAGTACATAGTACATAAAGCCATTTACCGTACATAGCACATTACAGTCAAATCCCTTCTCGTCCCCATGGATGACCCCCCTCAGATAGGGGTCCCTTGAC
Clone 1 PCR1                              L16055.....................................C............................................................................................T...............................H16218
Clone 2 PCR1                                    .....................................C............................................................................................................................
Clone 3 PCR1                                    .....................................C............................................................................................................................
Clone 4 PCR1                                    .............T........................................................C..................................................................T........................
```

```
Clone 5 PCR1                                    ........................................................................................................................................T.........................
Clone 6 PCR1                                    .....................................C..........T.........T....................................T..........................................C.......................
Clone 7 PCR1                                    .............T........................................................C...........................................................................................
Clone 1 PCR2                              L16055.............................................................................T.........................T..........................................................H16218
Clone 2 PCR2                                    .....................................C.....................................................................................................C......................
Clone 3 PCR2                                    .....................................C............................................................................................................................
Clone 4 PCR2                                    .....................................C............................................................................................................................
Clone 5 PCR2                                    .......................T.............C.................T...........................................................................T..............................
Clone 6 PCR2                                    .....................................C............................................................................................................................
Clone 7 PCR2                                    .....................................C............................................................................................................................
Clone 8 PCR2                                    .....................................C............................................................................................................................
Clone 9 PCR2                                    .....................................T............................................................................................................................
Clone 10 PCR2                                   .....................................C............................................................................................................................
Clone 11 PCR2                                   .....................................C............................................................................................................................
Clone 12 PCR2                                   .....................................C.....T....T..........................................T......................................................................
Clone 13 PCR2                                   .....................................C............................................................................................................................
Clone 14 PCR2                                   ......................................................................C...........................................................................................
Clone 1 PCR3                                                                                                                                                                    L16185.............................................................................................................................C..................................................................H16378
Clone 2 PCR3                                                                                                                                                                          ......................................................................................................................C.........................................................................
Clone 3 PCR3                                                                                                                                                                          ...................................TT.C....................T....T......T..T.................T................................C..................................................................
Clone 4 PCR3                                                                                                                                                                          ......................................................................................................................C.........................................................................
Clone 5 PCR3                                                                                                                                                                          ......................................C.................................................................T....................C..................................................................
Clone 6 PCR3                                                                                                                                                                          ......................................C......................................................................................C..................................................................
Clone 7 PCR3                                                                                                                                                                          ......T............................T..................................................................................C.........................................................................
Clone 8 PCR3                                                                                                                                                                          ..............................G.......C......................................................................................C...................................................T..............
Clone 9 PCR3                                                                                                                                                                          ......................................C......................................................................................C..................................................................
Clone 1 PCR4                                                                                                                                                                    L16185......................................C......................................................................................C..................................................................H16378
Clone 2 PCR4                                                                                                                                                                          ......................................CT..T..............................................................T....T..............C..................................................................
Clone 3 PCR4                                                                                                                                                                          ......................................C..............T.......................................................................C..................................................................
Clone 4 PCR4                                                                                                                                                                          ...........................A..........C..........................T....TT.T.T....................................................................................................................
Clone 5 PCR4                                                                                                                                                                          ......................................C......................................................................................C..................................................................
Clone 6 PCR4                                                                                                                                                                          ......................................C......................................................................................C..................................................................
Clone 7 PCR4                                                                                                                                                                          .......T........................T.....C.............................................................T....T..T................C..................................................................
Clone 8 PCR4                                                                                                                                                                          ......................................C.........................................................................................................................................................
Clone 9 PCR4                                                                                                                                                                          ......T..................T..T...T.....C.......................T.................................T............................C......................................................T...........
Clone 10 PCR4                                                                                                                                                                         ......................................C.............................................................T........................C................................................................A.
Clone 11 PCR4                                                                                                                                                                         ......................................C......................................................................................C........T......T.........T.T....T.................................
Clone 12 PCR4                                                                                                                                                                         ......................................C...........................................T.....................T....................C..................................................................
Clone 13 PCR4                                                                                                                                                                         ...................................T..C......................................................................................C..................................................................
Clone 14 PCR4                                                                                                                                                                         ......................................C......................................................................................C..................................................................
Clone 15 PCR4                                                                                                                                                                         .............................................................................................................................C.........................T........................................
Clone 16 PCR4                                                                                                                                                                         ......................................C......................................................................................C..................................................................
```

Mirador18 CRS

```
               111111111111111111111111111111111111111111111111111111111111111111111111111111111111111111111111111111111111111111111111111111111111111111111111111111111111111111111111111111111111111111111111111111111111111111111111111111111111111111111111111111111111111111111111111111111111111111111111111111111111111111111111111111111111111111111111111111111111111111111111111111111111111111
               666666666666666666666666666666666666666666666666666666666666666666666666666666666666666666666666666666666666666666666666666666666666666666666666666666666666666666666666666666666666666666666666666666666666666666666666666666666666666666666666666666666666666666666666666666666666666666666666666666666666666666666666666666666666666666666666666666666666666666666666666666666666666666
               000000000000000000000000000000000000000000000000000000000000000000000000000001111111111111111111111111111111111111111111111111111111111111111111111111111111111111111111111111111111111111111111111111111111111111111111111111111111111111111111111111111111111111111111111111111111111111111111111111111111111111111111111111111111111111111111111111111111111111111111111111111111111114
               222222233333333334444444444555555555566666666667777777777888888888899999999990000000000111111111122222222223333333333444444444455555555556666666666777777777788888888889999999999000000000011111111112222222222333333333344444444445555555555666666666677777777778888888888999999999900000000001111111111222222222233333333334444444444555555555566666666667777777777888888888899999999990
               3456789012345678901234567890123456789012345678901234567890123456789012345678901234567890123456789012345678901234567890123456789012345678901234567890123456789012345678901234567890123456789012345678901234567890123456789012345678901234567890123456789012345678901234567890123456789012345678901234567890123456789012345678901234567890123456789012345678901234567890123456789012345678901234567890 PCR1
               GTTCTTTCATGGGGAAGCAGATTTGGGTACCACCCAAGTATTGACTCACCCATCAACAACCGCTATGTATTTCGTACATTACTGCCAGCCACCATGAATATTGTACGGTACCATAAATACTTGACCACCTGTAGTACATAAAAACCCAATCCACATCAAAACCCCCTCCCCATGCTTACAAGCAAGTACAGCAATCAACCCTCAACTATCACACATCAACTGCAACTCCAAAGCACCCCTCACCCACTAGGATACCAACAAACCTACCCACCCTTAACAGTACATAGTACATAAAGCCATTTACCGTACATAGCACATTACAGTCAAATCCCTTCTCGTCCCCATGGATGACCCCCCTCAGATAGGGGTCCCTTGAC
Clone 1 PCR1                              L16055.....................................................................................................................T............................................H16218
Clone 2 PCR1                                    ..................................................................................................................................................................
Clone 3 PCR1                                    .....................................C..............................................................................................T.............................
Clone 4 PCR1                                    ..................................................................................................................................................................
Clone 5 PCR1                                    ..................................................................................................................................................................
Clone 6 PCR1                                    ..................................................................................................................................................................
Clone 7 PCR1                                    ..................................................................................................................................................................
Clone 8 PCR1                                    ..................................................................................................................................................................
Clone 1 PCR2                                                                                                                                                                    L16185................................................................................................................................................................................................H16378
Clone 2 PCR2                                                                                                                                                                          ................................................................................................................................................................................................
Clone 3 PCR2                                                                                                                                                                          ................................................................................................................................................................................................
Clone 4 PCR2                                                                                                                                                                          .....................................T................................................................................C.........................................................................
Clone 5 PCR2                                                                                                                                                                          ......................................................................................................................C.........................................................................
Clone 6 PCR2                                                                                                                                                                          ....................................................................................T.................................C.........................................................................
Clone 7 PCR2                                                                                                                                                                          .........................................................................T......................................................................................................................
Clone 8 PCR2                                                                                                                                                                          ................................................................................................................................................................................................
Clone 9 PCR2                                                                                                                                                                          ................................................................................................................................................................................................
Clone 1 PCR3                                                                                                                                                                    L16185................................................................................................................................................................................................H16378
Clone 2 PCR3                                                                                                                                                                          ................................................................................................................................................................................................
Clone 3 PCR3                                                                                                                                                                          .....................................C......................................................................................C............................................G......................
Clone 4 PCR3                                                                                                                                                                          ............................................................................................................................C...................................................................
Clone 5 PCR3                                                                                                                                                                          ................................................................................................................................................................................................
Clone 6 PCR3                                                                                                                                                                          ................................................................................................................................................................................................
Clone 7 PCR3                                                                                                                                                                          .....................................C......................................................................................C...................................................................
Clone 8 PCR3                                                                                                                                                                          ................................................................................................................................................................................................
Clone 9 PCR3                                                                                                                                                                          ............................................................................................................................................T...................................................
Clone 10 PCR3                                                                                                                                                                         ...............................................................T...................................................................................................................T............
Clone 11 PCR3                                                                                                                                                                         ...............................................................................................................C................................................................................
Clone 12 PCR3                                                                                                                                                                         ..............................................................................................................................................................C.................................
Clone 13 PCR3                                                                                                                                                                         ........................T.....................................................G.....G................................A..........................................................................
Clone 14 PCR3                                                                                                                                                                         ................................................................................................................................................................................................
Clone 15 PCR3                                                                                                                                                                         ............................................................................A..........................................................................G........................................
```

Mirador19 CRS

```
               111111111111111111111111111111111111111111111111111111111111111111111111111111111111111111111111111111111111111111111111111111111111111111111111111111111111111111111111111111111111111111111111111111111111111111111111111111111111111111111111111111111111111111111111111111111111111111111111111111111111111111111111111111111111111111111111111111111111111111111111111111111111111111
               666666666666666666666666666666666666666666666666666666666666666666666666666666666666666666666666666666666666666666666666666666666666666666666666666666666666666666666666666666666666666666666666666666666666666666666666666666666666666666666666666666666666666666666666666666666666666666666666666666666666666666666666666666666666666666666666666666666666666666666666666666666666666666
               000000000000000000000000000000000000000000000000000000000000000000000000000001111111111111111111111111111111111111111111111111111111111111111111111111111111111111111111111111111111111111111111111111111111111111111111111111111111111111111111111111111111111111111111111111111111111111111111111111111111111111111111111111111111111111111111111111111111111111111111111111111111111114
               222222233333333334444444444555555555566666666667777777777888888888899999999990000000000111111111122222222223333333333444444444455555555556666666666777777777788888888889999999999000000000011111111112222222222333333333344444444445555555555666666666677777777778888888888999999999900000000001111111111222222222233333333334444444444555555555566666666667777777777888888888899999999990
               3456789012345678901234567890123456789012345678901234567890123456789012345678901234567890123456789012345678901234567890123456789012345678901234567890123456789012345678901234567890123456789012345678901234567890123456789012345678901234567890123456789012345678901234567890123456789012345678901234567890123456789012345678901234567890123456789012345678901234567890123456789012345678901234567890 PCR1
               GTTCTTTCATGGGGAAGCAGATTTGGGTACCACCCAAGTATTGACTCACCCATCAACAACCGCTATGTATTTCGTACATTACTGCCAGCCACCATGAATATTGTACGGTACCATAAATACTTGACCACCTGTAGTACATAAAAACCCAATCCACATCAAAACCCCCTCCCCATGCTTACAAGCAAGTACAGCAATCAACCCTCAACTATCACACATCAACTGCAACTCCAAAGCACCCCTCACCCACTAGGATACCAACAAACCTACCCACCCTTAACAGTACATAGTACATAAAGCCATTTACCGTACATAGCACATTACAGTCAAATCCCTTCTCGTCCCCATGGATGACCCCCCTCAGATAGGGGTCCCTTGAC
Clone 1 PCR1                              L16055..................................................................................................................................................................H16218
Clone 2 PCR1                                    .....................................C............................................................................................................................
Clone 3 PCR1                                    .........................................C...............................................................................................T........................
Clone 4 PCR1                                    ..................................................................................................................................................................
Clone 5 PCR1                                    .........................................................................................................................................T........................
Clone 6 PCR1                                    ..................................................................................................................................................................
Clone 7 PCR1                                    ..................................................................................................................................................................
Clone 8 PCR1                                    ........................................................................................................................T.........................................
Clone 9 PCR1                                    ..............................................................................................T...................................................................
Clone 10 PCR1                                   ..................................................................................................................................................................
Clone 11 PCR1                                   .............T................................................................................T..................T...............TT...............................
Clone 12 PCR1                                   ..................................................................................................................................................................
Clone 13 PCR1                                   .....................................C............................................................................................................................
Clone 1 PCR2                                                                                                                                                                    L16185............................................................................................T......................T............................................................................H16378
Clone 2 PCR2                                                                                                                                                                          ....T......T...........................T................T....................................T..................................................................................................
Clone 3 PCR2                                                                                                                                                                          ............................................................................................T.........................................T.........................................................
Clone 4 PCR2                                                                                                                                                                          ......T.............................................................................T.................................C.........................................................................
Clone 5 PCR2                                                                                                                                                                          ............................................................................................T...................................................................................................
Clone 6 PCR2                                                                                                                                                                          ......................................................................................................................................................................................C.........
Clone 7 PCR2                                                                                                                                                                          ......................................C........................................G................................................................................................................
Clone 8 PCR2                                                                                                                                                                          ......................................C......................................................................................C.......................G..........................................
Clone 9 PCR2                                                                                                                                                                          ............................................................................................................T.T...........................C.....................................................
Clone 10 PCR2                                                                                                                                                                         ............................................................................................................T.........C.........................................................................
Clone 11 PCR2                                                                                                                                                                         ......................................C......................................................................................C..................................................................
Clone 12 PCR2                                                                                                                                                                         ......................................C........................................................................................................................................................T
Clone 13 PCR2                                                                                                                                                                         .................G....................C......................................................................................C..................................................................
Clone 14 PCR2                                                                                                                                                                         ............................................................................................T...................................................................................................
Clone 15 PCR2                                                                                                                                                                         .......T................................................................................................................................................................................A..A....
Clone 16 PCR2                                                                                                                                                                         .............................................................................................................................C..................................................................
Clone 17 PCR2                                                                                                                                                                         ..................................................................................................................................................T....T........................................
```

```
Clone 18 PCR2                                                                                                                                                                                  ...........................................................................................T...................................................................................................
Clone 19 PCR2                                                                                                                                                                                  .....T..................................................................T............................................C.........................................................................

Mirador20 069 126 193 274 278 ??? J1+16193*/J2b*

               111111111111111111111111111111111111111111111111111111111111111111111111111111111111111111111111111111111111111111111111111111111111111111111111111111111111111111111111111111111111111111111111111111111111111111111111111111111111111111111111111111111111111111111111111111111111111111111111111111111111111111111111111111111111111111111111111111111111111111111111111111111
               666666666666666666666666666666666666666666666666666666666666666666666666666666666666666666666666666666666666666666666666666666666666666666666666666666666666666666666666666666666666666666666666666666666666666666666666666666666666666666666666666666666666666666666666666666666666666666666666666666666666666666666666666666666666666666666666666666666666666666666666666666666
               000000000000000000000000000000000000000000000000000000000000000000000000000001111111111111111111111111111111111111111111111111111111111111111111111111111111111111111111111111111111111111111222222222222222222222222222222222222222222222222222222222222222222222222222222222222222222222222222222223333333333333333333333333333333333333333333333333333333333333333333333333333333333333333333333334
               222222233333333334444444444555555555566666666667777777777888888888899999999990000000000111111111122222222223333333333444444444455555555556666666666777777777788888888889999999999000000000011111111112222222222333333333344444444445555555555666666666677777777778888888888999999999900000000001111111111222222222233333333334444444444555555555566666666667777777777888888888899999999990
               345678901234567890123456789012345678901234567890123456789012345678901234567890123456789012345678901234567890123456789012345678901234567890123456789012345678901234567890123456789012345678901234567890123456789012345678901234567890123456789012345678901234567890123456789012345678901234567890123456789012345678901234567890123456789012345678901234567890123456789012345678901234567890 PCR1
               GTTCTTTCATGGGGAAGCAGATTTGGGTACCACCCAAGTATTGACTCACCCATCAACAACCGCTATGTATTTCGTACATTACTGCCAGCCACCATGAATATTGTACGGTACCATAAATACTTGACCACCTGTAGTACATAAAAACCCAATCCACATCAAAACCCCCTCCCCATGCTTACAAGCAAGTACAGCAATCAACCCTCAACTATCACACATCAACTGCAACTCCAAAGCCACCCCTCACCCACTAGGATACCAACAAACCTACCCACCCTTAACAGTACATAGTACATAAAGCCATTTACCGTACATAGCACATTACAGTCAAATCCCTTCTCGTCCCCATGGATGACCCCCCTCAGATAGGGGTCCCTTGAC
Clone 1 PCR1                             L16055.............T...........................................................................................................................T........................H16218
Clone 2 PCR1                                   .............T........................................................C..................................................................T........................
Clone 3 PCR1                                   ......................................................................C..A........................................................................................
Clone 4 PCR1                                   .............T........................................................C..................................................................T........................
Clone 5 PCR1                                   .....................................C............................................................................................................................
Clone 6 PCR1                                   .............T........................................................C..................................................................T........................
Clone 7 PCR1                                   .............T........................................................C..................................................................T........................
Clone 8 PCR1                                   .............T........................................................C..................................................................T........................
Clone 9 PCR1                                   .............T........................................................C..................................................................T........................
Clone 10 PCR1                                  .............T...........................................................................................................................T........................
Clone 11 PCR1                                  .............T........................................................C..................................................................T........................
Clone 12 PCR1                                  ......................................................................C..................................................................T........................
Clone 13 PCR1                                  .............T........................................................C..................................................................T........................
Clone 14 PCR1                                  .............T........................................................C..................................................................T........................
Clone 15 PCR1                                  .............T........................................................C..................................................................T........................
Clone 1 PCR2                                                                                                                                                                             L16185......T................................................................................A...T........................................A..........................................................H16378
Clone 2 PCR2                                                                                                                                                                                   ......T................................................................................A...T.....................................................................T.............................
Clone 3 PCR2                                                                                                                                                                                   .....T.............................................................................T...........................................................................................................
Clone 4 PCR2                                                                                                                                                                                   ......T................................................................................A...T...................................................................................................
Clone 5 PCR2                                                                                                                                                                                   ......T................................................................................A...T...................................................................................................
Clone 6 PCR2                                                                                                                                                                                   ......T................................................................................A...T...................................................................................................
Clone 1 PCR3                                                                                                                                                                             L16185......T................................................................................A...T...................................................................................................H16378
Clone 2 PCR3                                                                                                                                                                                   ......T................................................................................A...T...................................................................................................
Clone 3 PCR3                                                                                                                                                                                   .....T.............................................................................T................................................A..........................................................
Clone 4 PCR3                                                                                                                                                                                   ......T................................................................................A...T...................................................................................................
Clone 5 PCR3                                                                                                                                                                                   ......T................................................................................A...T...................................................................................................
Clone 6 PCR3                                                                                                                                                                                   ......T................................................................................A...T...................................................................................................
Clone 7 PCR3                                                                                                                                                                                   ......T................................................................................A...T...................................................................................................
Clone 8 PCR3                                                                                                                                                                                   ......T................................................................................A...T...................................................................................................
Clone 9 PCR3                                                                                                                                                                                   ......T................................................................................A...T...................................................................................................
Clone 10 PCR3                                                                                                                                                                                  ......T................................................................................A...T...................................................................................................
Clone 11 PCR3                                                                                                                                                                                  ......T................................................................................A...T...................................................................................................
Clone 12 PCR3                                                                                                                                                                                  ......T................................................................................A...T...................................................................................................
Clone 13 PCR3                                                                                                                                                                                  ......T................................................................................A...T...................................................................................................
Clone 14 PCR3                                                                                                                                                                                  ...............................................................T................................G.........G....................................................................................
Clone 15 PCR3                                                                                                                                                                                  ......T................................................................................A...T...................................................................................................
Clone 16 PCR3                                                                                                                                                                                  ......T................................................................................A...T.............................................................................C.....................

Mirador22 224 311 K*

               111111111111111111111111111111111111111111111111111111111111111111111111111111111111111111111111111111111111111111111111111111111111111111111111111111111111111111111111111111111111111111111111111111111111111111111111111111111111111111111111111111111111111111111111111111111111111111111111111111111111111111111111111111111111111111111111111111111111111111111111111111111
               666666666666666666666666666666666666666666666666666666666666666666666666666666666666666666666666666666666666666666666666666666666666666666666666666666666666666666666666666666666666666666666666666666666666666666666666666666666666666666666666666666666666666666666666666666666666666666666666666666666666666666666666666666666666666666666666666666666666666666666666666666666
               000000000000000000000000000000000000000000000000000000000000000000000000000001111111111111111111111111111111111111111111111111111111111111111111111111111111111111111111111111111111111111111222222222222222222222222222222222222222222222222222222222222222222222222222222222222222222222222222222223333333333333333333333333333333333333333333333333333333333333333333333333333333333333333333333334
               222222233333333334444444444555555555566666666667777777777888888888899999999990000000000111111111122222222223333333333444444444455555555556666666666777777777788888888889999999999000000000011111111112222222222333333333344444444445555555555666666666677777777778888888888999999999900000000001111111111222222222233333333334444444444555555555566666666667777777777888888888899999999990
               345678901234567890123456789012345678901234567890123456789012345678901234567890123456789012345678901234567890123456789012345678901234567890123456789012345678901234567890123456789012345678901234567890123456789012345678901234567890123456789012345678901234567890123456789012345678901234567890123456789012345678901234567890123456789012345678901234567890123456789012345678901234567890 PCR1
               GTTCTTTCATGGGGAAGCAGATTTGGGTACCACCCAAGTATTGACTCACCCATCAACAACCGCTATGTATTTCGTACATTACTGCCAGCCACCATGAATATTGTACGGTACCATAAATACTTGACCACCTGTAGTACATAAAAACCCAATCCACATCAAAACCCCCTCCCCATGCTTACAAGCAAGTACAGCAATCAACCCTCAACTATCACACATCAACTGCAACTCCAAAGCCACCCCTCACCCACTAGGATACCAACAAACCTACCCACCCTTAACAGTACATAGTACATAAAGCCATTTACCGTACATAGCACATTACAGTCAAATCCCTTCTCGTCCCCATGGATGACCCCCCTCAGATAGGGGTCCCTTGAC
Clone 1 PCR1                             L16055..................................................................................................................................................................H16218
Clone 2 PCR1                                   ..................................................................................................................................................................
Clone 3 PCR1                                   ..................................................................................................................................................................
Clone 4 PCR1                                   .............................................................C....................................................................................................
Clone 5 PCR1                                   ..................................................................................................................................................................
Clone 6 PCR1                                   ..................................................................................................................................................................
Clone 7 PCR1                                   ..................................................................................................................................................................
Clone 8 PCR1                                   ..................................................................................................................................................................
Clone 9 PCR1                                   ..................................................................................................................................................................
Clone 10 PCR1                                  ...............................................................................................................................C...T.C..T.........................
Clone 11 PCR1                                  ..................................................................................................................................................................
Clone 12 PCR1                                  ......................................................................................................................T...........................................
Clone 13 PCR1                                  ..............G..........................................................A........................................................................................
Clone 14 PCR1                                  ...........................................................................................................G......................................................
Clone 15 PCR1                                  ..................................................................................................................................................................
Clone 16 PCR1                                  .............................T.G..........................T.......................................................................................................
Clone 1 PCR2                                                                                                                                                                             L16185.....................................C.......................................................................T..............C..................................................................H16378
Clone 2 PCR2                                                                                                                                                                                   .....................................C......................................................................................C..................................................................
Clone 3 PCR2                                                                                                                                                                                   .....................................C.....................................................................T................C.............................................C....................
Clone 4 PCR2                                                                                                                                                                                   .....................................C.....................................................................T................C..................................................................
Clone 5 PCR2                                                                                                                                                                                   .....................................C....................................T.................................................C....................T.............................................
Clone 6 PCR2                                                                                                                                                                                   .....T.............................................................................T.................................C.........................................................................
Clone 7 PCR2                                                                                                                                                                                   .....................................C....................................T.................................................C....................T...................C.........................
Clone 8 PCR2                                                                                                                                                                                   .....................................C........................................G.............................................C..................................................................
Clone 9 PCR2                                                                                                                                                                                   T....................................C.........................................................T............................................T.........T........................................
Clone 10 PCR2                                                                                                                                                                                     ...........................C...........C........................................G.......T...................................................................................................
Clone 11 PCR2                                                                                                                                                                                  T....................................C.........................................................T............................................T.........T........................................
Clone 12 PCR2                                                                                                                                                                                  .....................................C...T..........T.........................G.........G...................................C..................................................................
Clone 1 PCR3                                                                                                                                                                             L16185.....................................C......................................................................................C..........................................T........................H16378
Clone 2 PCR3                                                                                                                                                                                   .....................................C......................................................................................C...................................................................
Clone 3 PCR3                                                                                                                                                                                   .....................................C......................................................................................C...................................................................
Clone 4 PCR3                                                                                                                                                                                   .....................................C......................................................................................C...................................................................
Clone 5 PCR3                                                                                                                                                                                   .....T............................T................................................T.................................C......C...................................................................
Clone 6 PCR3                                                                                                                                                                                   ...................................................................................T.................................C..........................................................................
Clone 7 PCR3                                                                                                                                                                                   .....................................C....................T.................................................................C...................................................................
Clone 8 PCR3                                                                                                                                                                                   ............................................................................................................................C.T....G............................................................
Clone 9 PCR3                                                                                                                                                                                   ..........T..........................C.......................T.................................T...TT.......................C.T.............TT..................................................
Clone 10 PCR3                                                                                                                                                                                  .....................................C......................................................................................C...................................................................
Clone 11 PCR3                                                                                                                                                                                  ............................................................................................................................C...................................................................
Clone 12 PCR3                                                                                                                                                                                  ......T................................................................................A...T.........................................................................................G..........
Clone 13 PCR3                                                                                                                                                                                  .....................................C......................................................................................C...................................................................
Clone 14 PCR3                                                                                                                                                                                  .....................................................................................................................C..............................G...........................................
Clone 15 PCR3                                                                                                                                                                                  ...........CA..............................................................................................T.........C.......................................T...............................T..

Mirador25 343 U3*

               111111111111111111111111111111111111111111111111111111111111111111111111111111111111111111111111111111111111111111111111111111111111111111111111111111111111111111111111111111111111111111111111111111111111111111111111111111111111111111111111111111111111111111111111111111111111111111111111111111111111111111111111111111111111111111111111111111111111111111111111111111111
               666666666666666666666666666666666666666666666666666666666666666666666666666666666666666666666666666666666666666666666666666666666666666666666666666666666666666666666666666666666666666666666666666666666666666666666666666666666666666666666666666666666666666666666666666666666666666666666666666666666666666666666666666666666666666666666666666666666666666666666666666666666
               000000000000000000000000000000000000000000000000000000000000000000000000000001111111111111111111111111111111111111111111111111111111111111111111111111111111111111111111111111111111111111111222222222222222222222222222222222222222222222222222222222222222222222222222222222222222222222222222222223333333333333333333333333333333333333333333333333333333333333333333333333333333333333333333333334
               222222233333333334444444444555555555566666666667777777777888888888899999999990000000000111111111122222222223333333333444444444455555555556666666666777777777788888888889999999999000000000011111111112222222222333333333344444444445555555555666666666677777777778888888888999999999900000000001111111111222222222233333333334444444444555555555566666666667777777777888888888899999999990
               345678901234567890123456789012345678901234567890123456789012345678901234567890123456789012345678901234567890123456789012345678901234567890123456789012345678901234567890123456789012345678901234567890123456789012345678901234567890123456789012345678901234567890123456789012345678901234567890123456789012345678901234567890123456789012345678901234567890123456789012345678901234567890 PCR1
               GTTCTTTCATGGGGAAGCAGATTTGGGTACCACCCAAGTATTGACTCACCCATCAACAACCGCTATGTATTTCGTACATTACTGCCAGCCACCATGAATATTGTACGGTACCATAAATACTTGACCACCTGTAGTACATAAAAACCCAATCCACATCAAAACCCCCTCCCCATGCTTACAAGCAAGTACAGCAATCAACCCTCAACTATCACACATCAACTGCAACTCCAAAGCCACCCCTCACCCACTAGGATACCAACAAACCTACCCACCCTTAACAGTACATAGTACATAAAGCCATTTACCGTACATAGCACATTACAGTCAAATCCCTTCTCGTCCCCATGGATGACCCCCCTCAGATAGGGGTCCCTTGAC
Clone 1 PCR1                             L16055...................................................................................................................................T..............................H16218
Clone 2 PCR1                                   ..................................................................................................................................................................
Clone 3 PCR1                                   ........................................................................T.........................................................................................
Clone 4 PCR1                                   ..................................................................................................................................................................
Clone 5 PCR1                                   ..................................................................................................................................................................
Clone 6 PCR1                                   ...........T.T....................................................................................................................................................
Clone 1 PCR1                                   ......................................................................................................................................T...........................
Clone 2 PCR1                                   ..................................................................................................................................................................
Clone 3 PCR1                                   ...........................................T...........................................................T..........................................................
Clone 4 PCR1                                   .............................................................................................................................................................A....
Clone 5 PCR1                                   .............T........................................................C...........................................................................................
```

```
Clone 6 PCR1   .......................................T..................C...................................C.................................C.....C.............................
Clone 7 PCR1   ....................................................................................................................................................................
Clone 8 PCR1   ....................................................................................................................................................................
Clone 9 PCR1   ..............T........................................................C...................................................................T........................
Clone 10 PCR1  ....................................................................................................................................................................
Clone 11 PCR1  .........................................................................................................................................T..........................
Clone 12 PCR1  .......................................T..................C.....................................................................C.....C.............................
Clone 13 PCR1  ....................................................................................................................................................................
Clone 14 PCR1  ..............................................................................................................................................T.....................
Clone 15 PCR1  .......................................T..................C.............................................................C.......C.....C.............................
Clone 16 PCR1  ..................................................................................................................................T.................................
Clone 1 PCR2   L16185...............T...............................................................................................................................................G..........T.......................H16378
Clone 2 PCR2   .....................................................C.........................................................................................................G..................................
Clone 3 PCR2   ...............................................................................................................................................................G.................A................
Clone 4 PCR2   ...................................T...........................................................................................................................G..................................
Clone 5 PCR2   .................................................................................................................C................................................................................
Clone 6 PCR2   .....................................T................T.................T.TTT..................................................................................G..................................
Clone 7 PCR2   ...............................................................................................................................................................G..................................
Clone 8 PCR2   .................................................................................................................C................................................................................
Clone 9 PCR2   .......................................................................TT.T.TT.T...............................................................................G..................................
Clone 10 PCR2  ...................................T...........................................................................................................................G..................................
Clone 11 PCR2  .................................................................................................................C................................................................................
Clone 1 PCR3   L16185...............T....................T........................................................T.................................................................G..................................H16378
Clone 2 PCR3   .........................T.......................................................T.............................................................................G..................................
Clone 3 PCR3   ...............................................................................................................................................................G........................G.........
Clone 4 PCR3   ...............................................................................................................................................................G........................G-........
Clone 5 PCR3   ....................................TT...............................................................TT........................................................G....................A..T..........
Clone 6 PCR3   ...................................................T..T...................T....................................................................................G..................................
Clone 7 PCR3   ...........................................................A...................................................................................................G..................................
Clone 8 PCR3   .T.........T.AT................................................................................................................................................A..................................
Clone 9 PCR3   ...............................................................................................................................................................G..................................
Clone 10 PCR3  ...........................................................................................C..................................................T................G..................................
```

Mirador26 069 126 145 231 261 J2a1a*

```
               111111111111111111111111111111111111111111111111111111111111111111111111111111111111111111111111111111111111111111111111111111111111111111111111111111111111111111111111111111111111111111111111111111111111111111111111111111111111111111111111111111111111111111111111111111111111111111111111111111111111111111111111111111111111111111111111111111111111111111111111111111111111
               666666666666666666666666666666666666666666666666666666666666666666666666666666666666666666666666666666666666666666666666666666666666666666666666666666666666666666666666666666666666666666666666666666666666666666666666666666666666666666666666666666666666666666666666666666666666666666666666666666666666666666666666666666666666666666666666666666666666666666666666666666666666
               000000000000000000000000000000000000000000000000000000000000000000000000000001111111111111111111111111111111111111111111111111111111111111111111111111111111111111111111111111111111112222222222222222222222222222222222222222222222222222222222222222222222222222222222222222222222222222223333333333333333333333333333333333333333333333333333333333333333333333333333333333333333333333334
               222222233333333334444444444555555555566666666667777777777888888888899999999990000000000111111111122222222223333333333444444444455555555556666666666777777777788888888889999999999000000000011111111112222222222333333333344444444445555555555666666666677777777778888888888999999999900000000001111111111222222222233333333334444444444555555555566666666667777777777888888888899999999990
               3456789012345678901234567890123456789012345678901234567890123456789012345678901234567890123456789012345678901234567890123456789012345678901234567890123456789012345678901234567890123456789012345678901234567890123456789012345678901234567890123456789012345678901234567890123456789012345678901234567890123456789012345678901234567890123456789012345678901234567890123456789012345678901234567890 PCR1
               GTTCTTTCATGGGGAAGCAGATTTGGGTACCACCCAAGTATTGACTCACCCATCAACAACCGCTATGTATTTCGTACATTACTGCCAGCCACCATGAATATTGTACGGTACCATAAATACTTGACCACCTGTAGTACATAAAAACCCAATCCACATCAAAACCCCCTCCCCATGCTTACAAGCAAGTACAGCAATCAACCCTCAACTATCACACATCAACTGCAACTCCAAAGCCACCCCTCACCCACTAGGATACCAACAAACCTACCCACCCTTAACAGTACATAGTACATAAAGCCATTTACCGTACATAGCACATTACAGTCAAATCCCTTCTCGTCCCCATGGATGACCCCCCTCAGATAGGGGTCCCTTGAC
Clone 1 PCR1   L16055..............T........................T...............................C..................A.........................................................................H16218
Clone 2 PCR1   .......................................................................C...................................................................T............N...........
Clone 3 PCR1   ..............T........................................................C......T......................................................T.T...........N.N..............
Clone 4 PCR1   .......................................................................C..................A........................................................NNN.N............
Clone 5 PCR1   ...........................................................................................................................................T........................
Clone 6 PCR1   ...................C.....................................................................................................T..........................................
Clone 7 PCR1   ..............T........................................................C..................A.........................................................................
Clone 8 PCR1   ..............T.........................A..............................C..................A.......................................T.................................
Clone 9 PCR1   .....................................C..................................................................................................T...........................
Clone 10 PCR1  ..............T........................................................C.............C.T..A.........................................................................
Clone 11 PCR1  ..............T........................................................C..................A.........................................................................
Clone 12 PCR1  ..............T......................C..............................................................................................................................
Clone 13 PCR1  .............................T.........................................C..................A.....................................C.....C.............................
Clone 1 PCR2   L16185..............................................C......................................T..................................C.........................................................................H16378
Clone 2 PCR2   .......T................................T..T..C.............................T.....................................................................................................................
Clone 3 PCR2   .......T.....................................................................................T....................................................................................................
Clone 4 PCR2   ........................................................................................................................C..............................G..........................................
Clone 5 PCR2   ..............................................C.........................................................................C..............................G..........................................
Clone 6 PCR2   ..............................................C.............................T.............................TT.TT..................................................................N................
Clone 7 PCR2   ..............................................C.......T.....................T...................................................................................................N.................
Clone 1 PCR3   L16185.............................T......T.........C.............................T.................................................................G.................................................T.H16378
Clone 2 PCR3   ..............................................C.............................T.....................................................................................................................
Clone 3 PCR3   ..............................................C.............................T.....................................................................................................................
Clone 4 PCR3   ..............................................C.............................T.....................................................................................................................
Clone 5 PCR3   ...............................................................................................................................C..................................................................
Clone 6 PCR3   ........................................................................................................................C.....................G........G..........................................
Clone 7 PCR3   ..............................................C.............................T.....................................................................................................................
Clone 8 PCR3   ..............................................C.............................T......................................G............................................................................T.
Clone 9 PCR3   .....................G.......................C.............................T.......................................................................................................................
Clone 10 PCR3  ..............................................C......................T......T.....................................................................................................................
Clone 11 PCR3  ..............................................C.............................T...................................................................................................A..A..............
```
