# Supplementary figures and images for: Mitochondrial DNA from El Mirador Cave (Atapuerca, Spain) Reveals the Heterogeneity of Chalcolithic Populations
Source: PLoS One. 2014 Aug 12;9(8):e105105. doi: 10.1371/journal.pone.0105105 (PMC4130614; doi:10.1371/journal.pone.0105105)

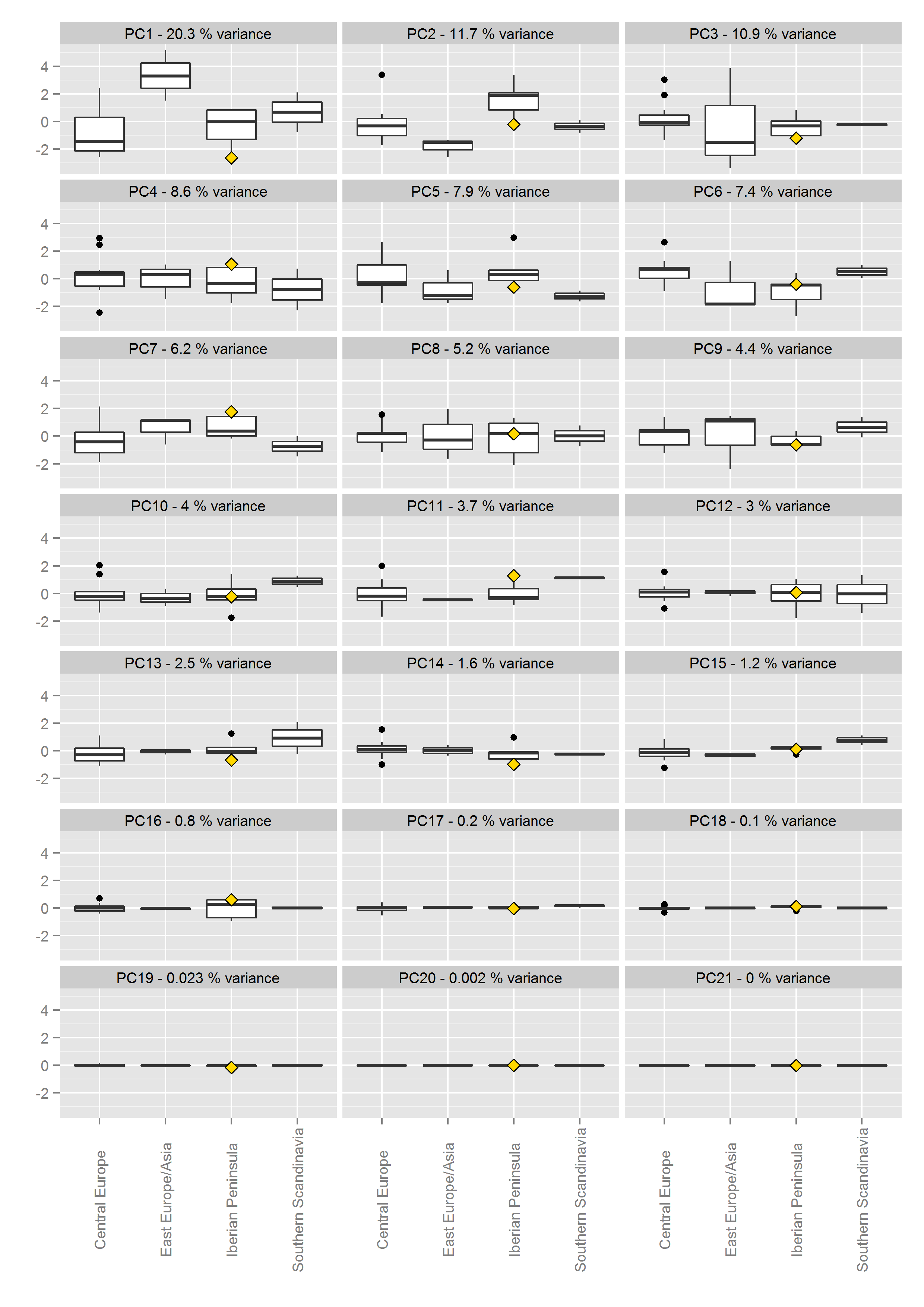

Supplement: Figure S2 — Boxplot grouped by geography of El Mirador principal components and other prehistoric cultures. PC1 and PC2 show the differentiation between geographical distributions of ancient cultures. El Mirador population (in yellow) shows affinities with prehistoric Central European cultures despite its location in Spain. (TIFF) [file pone.0105105.s002.tiff]

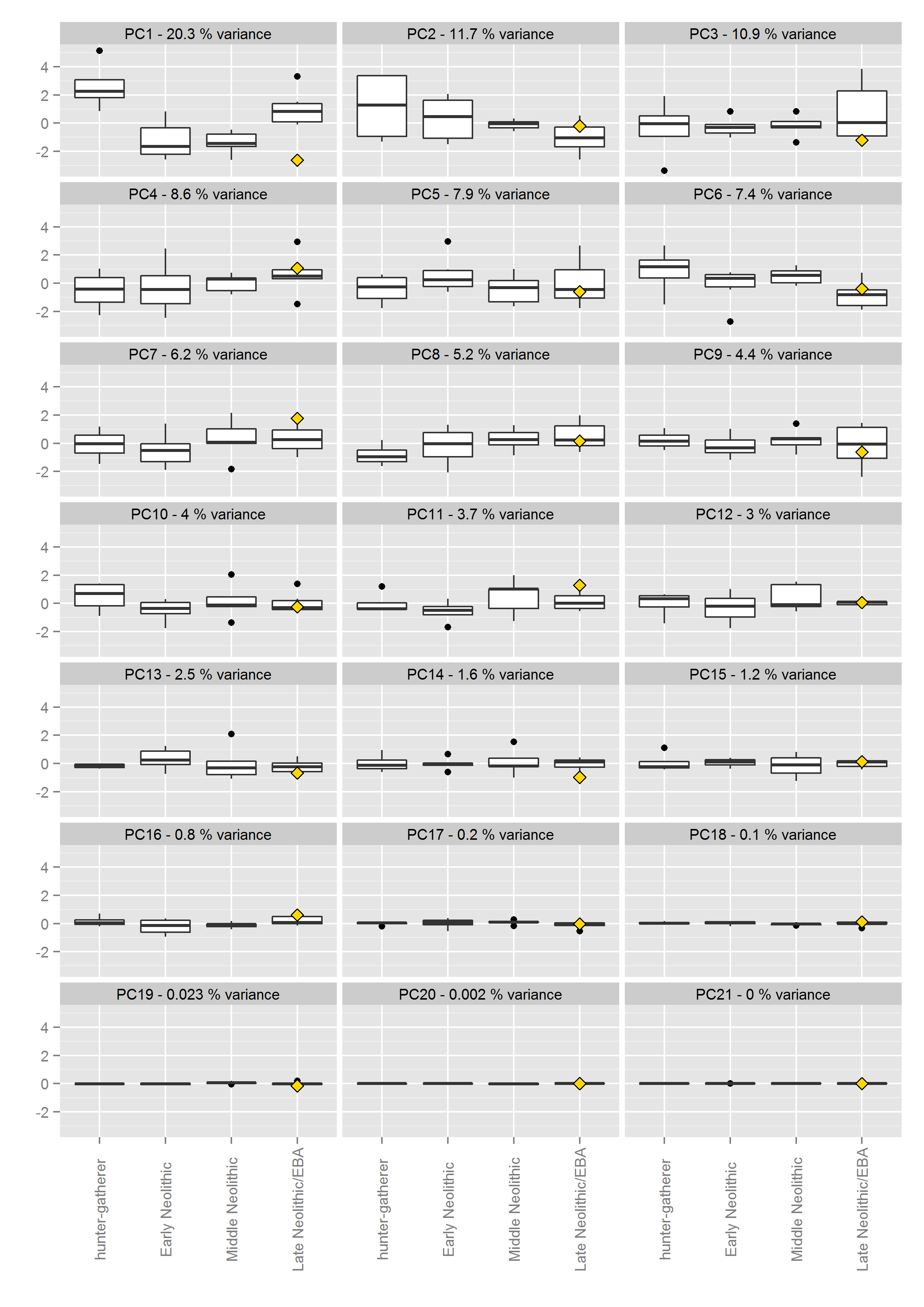

Supplement: Figure S3 — Boxplot grouped by chronology of El Mirador principal components and other prehistoric cultures. PC1 shows the differentiation between hunter-gatherer, Early/Middle Neolithic and Late Neolithic/EBA, whereas PC2 gives information for all the periods. El Mirador population (yellow) have traits of the Early/Middle Neolithic period in these components despite its Chalcolithic attribution. (TIFF) [file pone.0105105.s003.tiff]
